# National burden of hospitalized and non‐hospitalized influenza‐associated severe acute respiratory illness in Kenya, 2012‐2014

**DOI:** 10.1111/irv.12488

**Published:** 2017-12-15

**Authors:** Jeanette A. Dawa, Sandra S. Chaves, Bryan Nyawanda, Henry N. Njuguna, Carolyne Makokha, Nancy A. Otieno, Omu Anzala, Marc‐Alain Widdowson, Gideon O. Emukule

**Affiliations:** ^1^ College of Health Sciences Kenya AIDS Vaccine Institute (KAVI) ‐ Institute of Clinical Research University of Nairobi Nairobi Kenya; ^2^ Centers for Disease Control and Prevention Nairobi Kenya; ^3^ Influenza Division National Center for Immunization and Respiratory Diseases US Centers for Disease Control and Prevention Atlanta GA USA; ^4^ Kenya Medical Research Institute (KEMRI) Kisumu Kenya

**Keywords:** epidemiology, infection, pneumonia, virus

## Abstract

**Background:**

Influenza‐associated respiratory illness was substantial during the emergence of the 2009 influenza pandemic. Estimates of influenza burden in the post‐pandemic period are unavailable to guide Kenyan vaccine policy.

**Objectives:**

To update estimates of hospitalized and non‐hospitalized influenza‐associated severe acute respiratory illness (SARI) during a post‐pandemic period (2012‐2014) and describe the incidence of disease by narrow age categories.

**Methods:**

We used data from Siaya County Referral Hospital to estimate age‐specific base rates of SARI. We extrapolated these base rates to other regions within the country by adjusting for regional risk factors for acute respiratory illness (ARI), regional healthcare utilization for acute respiratory illness, and the proportion of influenza‐positive SARI cases in each region, so as to obtain region‐specific rates.

**Results:**

The mean annual rate of hospitalized influenza‐associated SARI among all ages was 21 (95% CI 19‐23) per 100 000 persons. Rates of non‐hospitalized influenza‐associated SARI were approximately 4 times higher at 82 (95% CI 74‐90) per 100 000 persons. Mean annual rates of influenza‐associated SARI were highest in children <2 years of age with annual hospitalization rates of 147 (95% CI of 134‐160) per 100 000 persons and non‐hospitalization rates of 469 (95% CI 426‐517) per 100 000 persons. For the period 2012‐2014, there were between 8153 and 9751 cases of hospitalized influenza‐associated SARI and 31 785‐38 546 cases of non‐hospitalized influenza‐associated SARI per year.

**Conclusions:**

The highest burden of disease was observed among children <2 years of age. This highlights the need for strategies to prevent influenza infections in this age group.

## 1 INTRODUCTION

Influenza is an important cause of respiratory illness in Kenya, accounting for up to 27% of all medically attended cases of respiratory illness.^1^ However, most Kenyan studies have focussed on providing subnational estimates of influenza disease burden.^2^, ^3^, ^4^, ^5^, ^6^, ^7^, ^8^, ^9^ The only national estimates of the incidence of influenza‐associated severe acute respiratory illness (SARI) were calculated for the period July 2009‐August 2011,^10^ when the influenza A(H1N1)pdm09 (H1N1pdm09) virus circulated at markedly high levels^11^ and presented with unusual epidemiology, affecting younger adults and sparing those ≥65 years of age.^12^ As H1N1pdm09 virus circulated in subsequent years, the severity and epidemiology of illness associated with the pandemic influenza strain could have changed, as has been observed for other pandemic viruses.^13^

Previous national estimates of the incidence of influenza‐associated SARI described the burden of severe influenza in children <5 years of age, and persons aged ≥5 years. From studies conducted in temperate countries, the risk of influenza is known to vary by age, being highest at extremes of age.^14^ Moreover, children <6 months of age are not eligible for vaccination and the World Health Organization (WHO) recommends vaccination for children 6‐23 months of age.^14^ Here, we adapted the methodologic approach used previously,^10^ to update estimates of the burden of hospitalized and non‐hospitalized influenza‐associated severe acute respiratory illness (SARI) during a non‐pandemic period (from 2012 through 2014), and describe the incidence of severe influenza disease by narrow age categories to include individuals <6 months of age, <2 years of age and ≥65 years of age which would be useful when considering interventions. The influenza vaccine is not included as part of the national immunization program in Kenya. Our results could inform Kenyan health authorities about the relative value of introducing influenza vaccination in specific age groups.

## 2 METHODS

We adapted a previously described method used to estimate the burden of severe influenza disease in Kenya.^10^ We calculated base rates of hospitalized SARI from Siaya County Referral Hospital (SCRH) in Nyanza region. These rates were extrapolated to the other regions in the country, by adjusting for regional risk factors for SARI and hospitalization. To calculate rates of non‐hospitalized SARI, non‐hospitalized SARI cases were indirectly estimated from healthcare utilization data on the proportion of acute respiratory illnesses that were hospitalized in Nyanza region. Region‐specific data on the proportion of influenza‐associated SARI were used to estimate regional rates of hospitalized and non‐hospitalized influenza‐associated SARI cases.

There were a few differences between our methodology and the methodology originally proposed by Fuller et al.^10^ A description of these differences and their possible impact on the results of the study, including the equations used in the analysis, have been described in detail in the Appendix S1.

### 2.1 Base site

SCRH is located in Nyanza region which has the highest HIV prevalence at 15%^15^ and the highest mortality among children aged <5 years within the country at 82 deaths per 1000 live births.^16^ SCRH is embedded in a Health and Demographic Surveillance System (HDSS) in Karemo division. The Karemo division of the HDSS has a population of approximately 80 000 inhabitants.^5^

We used SCRH and HDSS data from January 2012 through December 2014 as the basis for estimating the incidence of hospitalized SARI in the Nyanza region (henceforth referred to as base rates), which were then extrapolated to the other regions using the steps outlined further below.

### 2.2 Calculation of base rates of hospitalized SARI

Hospitalized SARI was clinically defined as a patient hospitalized with acute onset illness (within 14 days), reported fever (or recorded temperature of ≥38°C), and a cough. We calculated the annual age‐specific base rates of hospitalized SARI by dividing the age‐specific number of hospitalized SARI patients at SCRH who were enrolled in the HDSS, by the age‐specific population of Karemo residents within the HDSS. These base rates of hospitalized SARI, which were assumed to be representative of the Nyanza region, were calculated for each of the following age groups: 0‐5 months, 6‐11 months, 12‐23 months, 2‐4 years, 5‐14 years, 15‐49 years, 50‐64 years, and ≥65 years (Appendix S1: Equation 1).

### 2.3 Calculation of regional rates of hospitalized SARI

To obtain region‐specific rates of hospitalized SARI, we first calculated an adjustment factor for each of the other 7 regions in Kenya. Calculation of the adjustment factor used the population prevalence of region‐specific risk factors for SARI,^16^, ^17^, ^18^ the relative risk of each of the risk factors,^19^, ^20^, ^21^ and region‐specific healthcare‐seeking practices for acute respiratory illness (ARI)^22^ (Appendix S1: Equation 2).

For children <5 years old, 6 risk factors for SARI were used for adjustment: malnutrition (weight for age Z‐score ≤−2), low birthweight (<2500 g), non‐exclusive breastfeeding (during the first 4 months of life), household pollution (as indicated by use of solid fuels for cooking), crowding (≥5 persons in a household), and HIV prevalence. For individuals ≥5 years old, 3 SARI risk factors were used for adjustment: household air pollution, crowding, and HIV prevalence. The regional adjustment factor was multiplied by the base SARI rate to estimate the region‐specific hospitalized SARI rates (Appendix S1: Equation 3).

### 2.4 Calculation of regional rates of non‐hospitalized SARI

The rates of non‐hospitalized SARI were estimated using data on self‐reported episodes of pneumonia from a healthcare utilization survey (HUS) that was conducted in the Nyanza region.^22^ In the HUS, pneumonia was defined as reported episodes of acute respiratory illness (ARI) characterized by cough and difficulty breathing for more than 2 days, or a diagnosis of pneumonia by a healthcare worker within the last 12 months. For the purposes of this study, non‐hospitalized pneumonia as described in the HUS was equated to non‐hospitalized SARI. The definition of pneumonia in the HUS could have included cases of ARI that do not require hospitalization, and consequently, the definition of SARI in the current study differs from the WHO definition that all cases of SARI require hospitalization.^23^ The HUS provided data for 2 broad age groups: individuals <5 and ≥5 years of age.

As the HUS data were only available for the base region, we extrapolated the percentage of all those who reported pneumonia that resulted in hospitalization to the other regions by multiplying this percent by the proportion of individuals seeking healthcare for ARI for each region, compared to the base region (Appendix S1: Equation 5).

The total rates of regional SARI (combining hospitalized and non‐hospitalized SARI) in the community were calculated by multiplying the region‐specific rate of hospitalized SARI by the reciprocal of the estimated proportion of those with pneumonia who were hospitalized. In the final step, the rate of non‐hospitalized SARI was calculated as the difference of the overall SARI rates in the community and the rate of hospitalized SARI (Appendix S1: Equation 4).

### 2.5 Calculation of regional rates of hospitalized and non‐hospitalized influenza‐associated SARI

**Figure 1:**
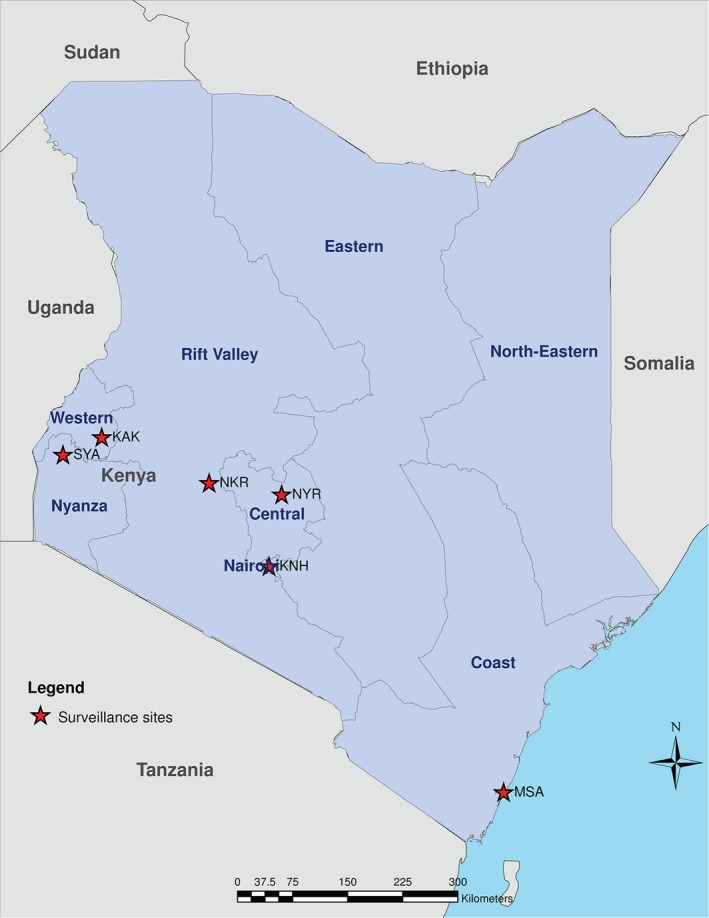
Map showing the location of the influenza surveillance sites

The proportion of SARI due to influenza was collected from six influenza surveillance sites spread across the country: SCRH representing Nyanza region, Kakamega County Referral Hospital (CRH) representing the Western region, Mombasa CRH representing the Coast region, Nakuru CRH representing the Rift Valley region, Nyeri CRH representing the Central region, and Kenyatta National Hospital (KNH) representing the Nairobi region (Figure 1). As surveillance data were not available in the Eastern and North Eastern regions during the study period, surveillance data from the sites in the neighboring regions were used to estimate the average proportion of hospitalized influenza‐associated SARI that was assigned to these two regions.

The region‐specific rates of hospitalized and non‐hospitalized influenza‐associated SARI were calculated by applying the proportion of SARI cases that tested positive for influenza virus in each site to the regional rates of both hospitalized and non‐hospitalized SARI (Appendix S1: Equation 6 and 7).

### 2.6 Calculation of the number of hospitalized and non‐hospitalized SARI cases

To obtain the number of cases, the region‐specific rates of hospitalized and non‐hospitalized SARI were multiplied with regional population data from the national census^18^ (Appendix S1: Equations 8 and 9). The national census data of 2009 were projected to the study period using the annual population growth rate.^24^ A similar approach was used to estimate the region‐specific number of hospitalized and non‐hospitalized influenza‐associated SARI cases (Appendix S1: Equations 10 and 11).

### 2.7 Data analysis

Data analysis was performed using Microsoft Excel, R version 3.3.1^25^ and Stata version 13.0.^26^ Confidence intervals (CIs) of the regional SARI rates were estimated by running 1000 iterations of each of the risk factors for hospitalized SARI (Table S1) by allowing the specific risk factor to vary within their 95% CI limits, while keeping all the other risk factors constant. The 2.5th and 97.5th values of these outputs were presented, respectively, as the lower and upper limits of the 95% CI of the regional hospitalized SARI rates. All rates were reported per 100 000 persons.

### 2.8 Ethical review

The study protocol at SCRH was approved by both the ethical review committee of the Kenya Medical Research Institute (KEMRI) (SSC‐1801), and the institutional review board (IRB) of the U.S. Centers for Disease Control and Prevention (CDC) (CDC‐3308). Surveillance for influenza within the other 5 influenza sentinel surveillance sites was part of routine surveillance by the Kenya Ministry of Health. Verbal consent to collect samples from patients at these sites was therefore considered adequate.

## 3 RESULTS

### 3.1 Rates of hospitalized and non‐hospitalized SARI

**Figure 2:**
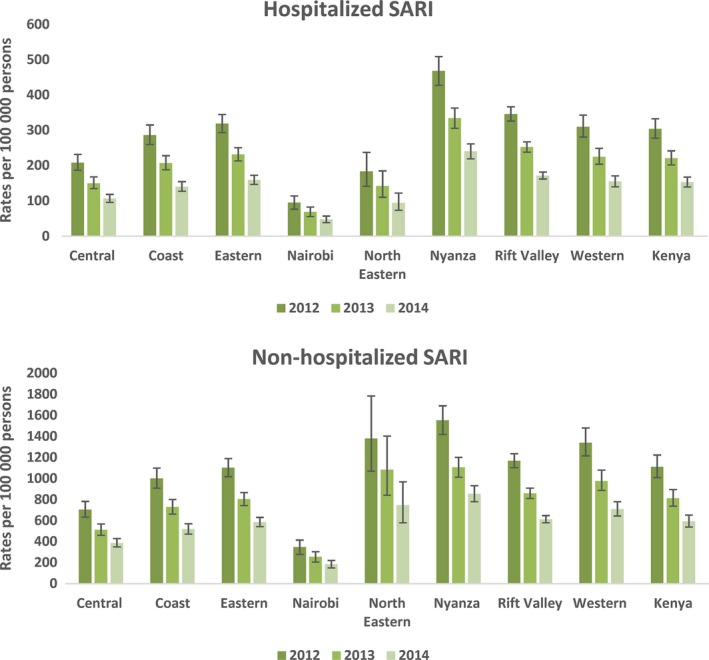
Rates of hospitalized and non‐hospitalized severe acute respiratory illness (SARI) per 100 000 population in Kenya by region, 2012 to 2014

The national mean annual rate of hospitalized SARI was 228.1 (95% CI 208.1‐249.4) per 100 000 persons (Table S2). Rates of hospitalized SARI were highest in Nyanza at 350.8 (95% CI 320.3‐381.2) per 100 000 persons and lowest in Nairobi at 71.6 (95% CI 57.3‐85.3) per 100 000 persons (Table S2). The national annual mean rate of non‐hospitalized SARI was 3.7 times higher than the rates of hospitalized SARI at 847.1 (95% CI 768.3‐932.0) per 100 000 persons (Table S3). Rates of non‐hospitalized SARI were highest in Nyanza at 1183.2 (95% CI 1079.3‐1285.7) per 100 000 persons and lowest in Nairobi at 265.6 (95% CI 212.7‐315.6) per 100 000 persons (Table S3). Rates of hospitalized and non‐hospitalized SARI were higher among children <5 years of age compared to the older age groups (Table S2 and S3). Rates of hospitalized and non‐hospitalized SARI were highest in 2012 and lowest in 2014 (Figure 2).

### 3.2 Rates of hospitalized influenza‐associated SARI

**Figure 3:**
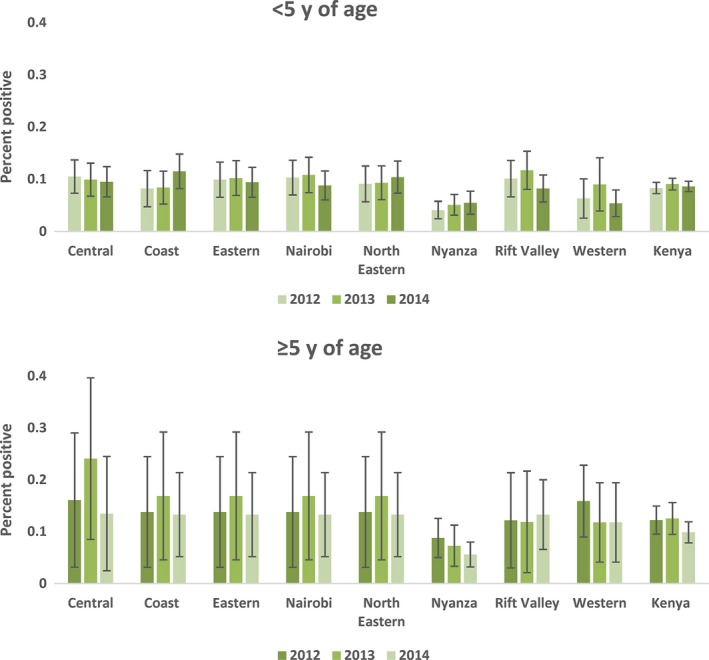
Proportion of severe acute respiratory infections (SARI) among children <5 years of age and individuals ≥5 years of age that tested positive for influenza by region, 2012 to 2014

**Figure 4:**
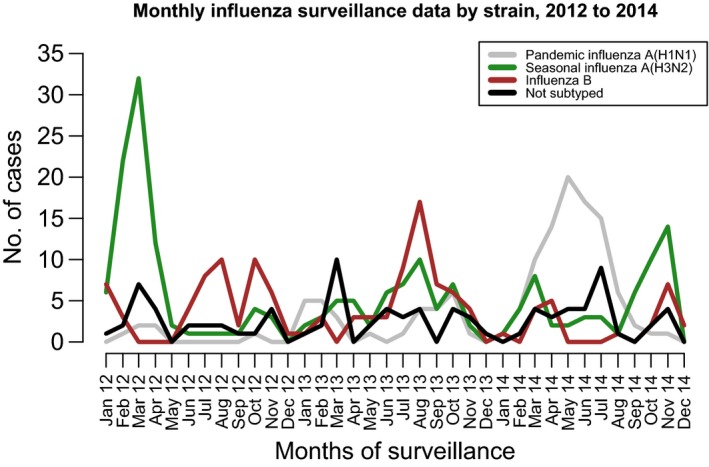
Monthly influenza‐positive cases by subtype, 2012 to 2014

Among children <5 years of age, the average annual influenza positivity was 8% in 2012 and 9% in 2013 and 2014, while among individuals ≥5 years of age, the average influenza positivity was 12% in 2012, 13% in 2013, and 10% in 2014 (Figure 3). The identified circulating influenza subtypes varied throughout the years (Figure 4). Influenza A(H3N2) virus accounted for 48% of viruses detected in 2012, influenza B accounted for 35% of influenza viruses detected in 2013, and H1N1pdm09 accounted for 48% of influenza viruses detected in 2014 (Table 1).

**Table 1:** Circulating influenza subtypes by year, 2012 to 2014

Year	2012		2013		2014	
Influenza subtype	Number	Percent	Number	Percent	Number	Percent
Influenza B	59	29.4	77	35.0	21	7.8
Pandemic influenza A(H1N1)	7	3.5	32	14.6	129	48.1
Seasonal influenza A(H3N2)	97	48.3	70	31.8	80	29.9
Not subtyped	38	18.9	41	18.6	38	14.2
Total	201	100.0	220	100.0	268	100.00

**Figure 5:**
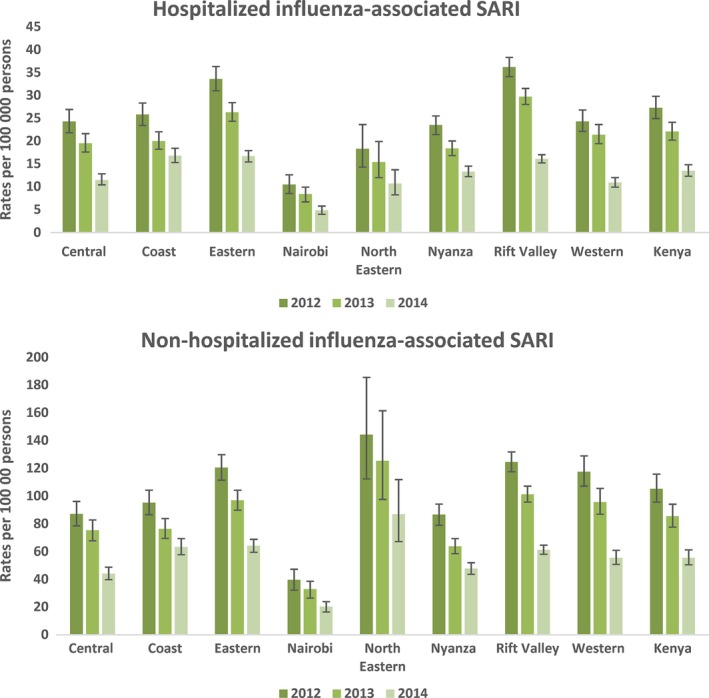
Rates of hospitalized and non‐hospitalized influenza‐associated severe acute respiratory illness (SARI) per 100 000 population in Kenya by region, 2012 to 2014

The national mean annual rate of hospitalized influenza‐associated SARI was 20.8 (95% CI 19.0‐22.7) per 100 000 persons (Table 2). Rates of hospitalized influenza‐associated SARI were highest in Rift Valley region (26.6 [95% CI 25.1‐28.2] per 100 000 persons) and lowest in Nairobi (7.8 [95% CI 6.3‐9.3] per 100 000 persons) (Table S4). Overall, rates of hospitalized influenza‐associated SARI were higher among children <5 years of age (100.6 [95% CI 91.7‐110.3] per 100 000 persons) than among individuals ≥5 years of age (6.3 [95% CI 5.8‐6.8] per 100 000 persons) (Table 2). The mean annual rates of hospitalized influenza‐associated SARI were highest among children <2 years of age at 146.6 (95% CI 133.8‐160.3) per 100 000 persons. Among the <2 years age group, rates among children 0‐5 months of age were 112.2 (95% CI 102.5‐122.6) per 100 000 persons, while rates among children 6‐11 months of age and 12‐23 months of age were 169.7 (95% CI 155.1‐ 185.8) per 100 000 persons and 152.7 (95% CI 139.3‐167.1) per 100 000 persons, respectively (Table 2). Among individuals ≥5 years of age, the mean annual rates of hospitalized influenza‐associated SARI were highest among persons aged ≥65 years (9.8 [95% CI 8.9‐10.5] per 100 000 population) and school age children 5‐14 years of age (9.4 [95% CI 8.7‐10.1] per 100 000 population) (Table 2). Rates of hospitalized influenza‐associated SARI were highest in 2012 and lowest in 2014 (Figure 5).

**Table 2:** Average annual national age‐specific rate and number of hospitalized and non‐hospitalized influenza‐associated severe acute respiratory illness (SARI) in Kenya, 2012 to 2014

Age	Hospitalized SARI cases		Non‐hospitalized SARI cases	
	Rate^a^	No. of cases	Rate^a^	No. of cases
<5 years	100.6 (91.7‐110.3)	6642 (6055‐7280)	325.7 (294.7‐360.3)	21 502 (19 451‐23 782)
<2 years	146.6 (133.8‐160.3)	3714 (3391‐4063)	469.0 (425.7‐516.6)	11 885 (10 787‐13 091)
0‐5 months	112.2 (102.5‐122.6)	755 (690‐825)	358.7 (326.0‐394.7)	2414 (2194‐2656)
6‐11 months	169.7 (155.1‐185.8)	1163 (1063‐1273)	542.6 (493.0‐598.1)	3718 (3378‐4098)
0‐11 months	141.2 (129.1‐154.5)	1918 (1753‐2098)	451.5 (410.3‐497.3)	6132 (5572‐6754)
12‐23 months	152.7 (139.3‐167.1)	1796 (1638‐1965)	489.2 (443.4‐538.8)	5753 (5215‐6337)
2‐4 years	72.0 (65.5‐79.1)	2928 (2664‐3217)	236.5 (213.0‐262.9)	9617 (8664‐10 691)
≥5 years	6.3 (5.8‐6.8)	2287 (2098‐2471)	37.3 (34.0‐40.7)	13 539 (12 334‐14 764)
5‐14 years	9.4 (8.7‐10.1)	1113 (1025‐1199)	56.7 (51.6‐61.8)	6695 (6103‐7299)
15‐49 years	4.1 (3.8‐4.5)	846 (772‐917)	24.1 (21.9‐26.3)	4939 (4485‐5387)
50‐64 years	7.3 (6.7‐8.0)	183 (169‐200)	42.4 (39.1‐46.7)	1066 (982‐1175)
≥65 years	9.8 (8.9‐10.5)	145 (132‐155)	56.7 (51.6‐61.0)	839 (764‐903)
All ages	20.8 (19.0‐22.7)	8929 (8153‐9751)	81.7 (74.1‐89.9)	35 041 (31 785‐38 546)

a Rates calculated per 100 000 persons.

### 3.3 Rates of non‐hospitalized influenza‐associated SARI

The mean annual rate of non‐hospitalized influenza‐associated SARI was 81.7 (95% CI 74.1‐89.9) per 100 000 persons (Table 2). Rates of non‐hospitalized influenza‐associated SARI were highest in the North Eastern region (120.2 [95% CI 94.0‐155.7] per 100 000 persons) and lowest in Nairobi region (30.5 [95%CI 24.4‐36.0] per 100 000 persons) (Table S5). Rates were higher among children <5 years of age (325.7 [95% CI 294.7‐360.3] per 100 000 persons) as compared to individuals aged ≥5 years of age (37.3 [95% CI 34.0‐40.7] per 100 000 persons) (Table 2). Similar to the hospitalized rates, rates of non‐hospitalized influenza‐associated SARI were highest among children <2 years of age (469.0 [95% CI 425.7‐516.6] per 100 000 persons) (Table 2). Among individuals aged ≥5 years, rates of non‐hospitalized influenza‐associated SARI were highest among children 5‐14 years of age (56.7 [95% CI 51.6‐61.8] per 100 000 persons) and the elderly ≥65 years of age (56.7 [95% CI 51.6‐61.0] per 100 000 persons) (Table 2). Rates of non‐hospitalized influenza‐associated SARI were also highest in 2012 and lowest in 2014 (Figure 5).

### 3.4 Number of hospitalized and non‐hospitalized influenza‐associated SARI cases

For the period 2012 through 2014, there were between 8153 and 9751 cases of hospitalized influenza‐associated SARI and 31 785‐38 546 cases of non‐hospitalized influenza‐associated SARI per year. Among children <5 years of age, the annual number of influenza‐associated SARI hospitalized cases was 6055‐7280 and of non‐hospitalized cases was 19 451‐23 782 (Table 2). Among those ≥5 years of age, the annual number of influenza‐associated SARI hospitalized cases was 2098‐2471 and of non‐hospitalized cases was 12 334‐14 764 (Table 2). Overall, there was a 30‐40% decrease in the number of influenza‐associated SARI cases during 2012‐2014 compared to previously estimated numbers from the 2009‐2011 period described in a study which used a similar methodology.^10^ There was a 66‐73% decline in the number of hospitalized influenza‐associated SARI cases, while the number of non‐hospitalized influenza‐associated SARI cases dropped 5‐15% during the same time frame (Table 3).

**Table 3:** Comparison of average annual national rate and number of hospitalized and non‐hospitalized influenza‐associated severe acute respiratory illness (SARI) cases in Kenya during and shortly after the 2009 influenza pandemic (2009‐2011) with seasonal influenza (2012‐2014)

Age group	Hospitalized SARI cases				Non‐hospitalized SARI cases			
	2009‐2011^a^	2012‐2014			2009‐2011^a^		2012‐2014	
	Rate^b^	No. of cases	Rate^b^	No. of cases	Rate^b^	No. of cases	Rate^b^	No. of cases
<5 years	290‐470	17 129‐7659	92‐110	6055‐7280	330‐510	19 798‐30 275	295‐360	19 451‐23 782
≥5 years	21‐24	6882‐7836	6‐7	2098‐2471	42‐47	13 592‐15 270	34‐41	12 334‐14 764
All ages	61‐90	24 011‐35 495	19‐23	8153‐9751	84‐115	33 390‐45 545	74‐90	31 785‐38 546

a Data from Fuller et al.^10^^.^

b Rate per 100 000 persons.

## 4 DISCUSSION

From 2012 through 2014, we estimated that the annual rate of hospitalized influenza‐associated SARI was 21 per 100 000 population, while the annual rate of non‐hospitalized influenza‐associated SARI was 4 times higher at 82 per 100 000 population. Despite regional and year‐to‐year variation on rates of influenza‐associated SARI, the group consistently most at risk for hospitalization was children <5 years of age, and especially those <2 years of age. Among older age groups, those 5‐14 years of age and adults ≥65 years of age had the highest hospitalization rates, though not in the same magnitude as that reported for young children.

Our findings support the hypothesis that the burden of influenza in Kenya during the influenza A(H1N1)pdm09 pandemic period (i.e. 2009 to 2010) was higher than during non‐pandemic years. Overall, there was a 30–40% decline in the number of influenza‐associated SARI cases between 2009‐2011 and 2012‐2014. In our study, the number of hospitalized influenza‐associated SARI cases was higher during 2012 when almost 50% of the samples were positive for H3N2 virus. H3N2 predominant seasons in the USA have been described as causing more severe disease in the very young and the very old.^27^ In contrast, in 2014 we saw a resurgence of the H1N1pdm09 virus strain, representing almost 50% of the samples tested. That year was the mildest in terms of influenza‐associated SARI hospitalizations. This may indicate a decline in the severity of the H1N1pdm09 virus strain over the years. The 2012‐2014 decline in hospitalized influenza‐associated SARI rates could also be attributed to a low threshold for admissions combined with increased healthcare seeking associated with heightened public awareness around the 2009 pandemic.

Based on the findings of the healthcare utilization survey, hospitalizations were estimated to occur in about 20% of influenza‐associated SARI cases. In keeping with this finding, a study conducted in rural Kenya estimated that approximately 10‐20% of patients with laboratory confirmed influenza‐associated pneumonia were hospitalized.^7^ Not all cases of influenza‐associated SARI require hospitalization, some cases may be managed in the outpatient setting; however, in countries with relatively low healthcare utilization rates such as Kenya, where only 48% of children <5 years of age and 34% of individuals ≥5 years of age with pneumonia reportedly seek medical care in hospitals,^22^ calculating the burden of disease based on SARI cases admitted to hospitals alone would grossly underestimate the true burden of influenza disease in the community.

Using a similar methodology, a South African study estimated rates of hospitalized influenza‐associated SARI among children <5 years of age of 58‐276 per 100 000 persons 28 which is comparable to our estimates of 92‐110 per 100 000 children. Rates of hospitalized influenza‐associated SARI among Kenyan children <5 years of age in 2012 and 2013 were approximately twice as high as the influenza‐associated hospitalization rates observed among the same age group in the United States.^29^ In fact, the average annual rate of influenza‐associated hospitalizations in our study for children <5 years was higher than that reported in European countries or the Americas for the same age group,^11^ underscoring the need to explore the cost‐effectiveness of influenza vaccines among those at highest risk of hospitalization.

Our rates of influenza‐associated hospitalization among those <2 years were twice as high as that among children 2‐4 years. Children <2 years of age are at high risk for severe influenza disease.^14^ Among the <2 years age group, rates of influenza‐associated hospitalization were estimated to be higher in the 6–23‐month age group than in the 0–5‐month age group. These findings are in contrast to the USA, where rates of influenza‐associated hospitalization are higher in the 0–5‐month age group than in the 6–23‐month age group^30^; however, these differences could reflect an increased likelihood of newborn hospitalizations in the USA for potential sepsis workup investigations. Few studies in Kenya have attempted to describe the incidence of hospitalized influenza‐associated SARI among very young children,^5^, ^31^ yet these findings have an important bearing on the implementation of influenza vaccine policies. While children 6‐23 months of age can be directly vaccinated against influenza, it is recommended that protection of children <6 months of age be provided through maternal vaccination.^14^

In contrast to the high rates of hospitalized influenza‐associated SARI among young children, rates for those ≥65 years of age were 9‐11 per 100 000 persons. The rates of influenza‐associated hospitalization among adults ≥65 years observed in the USA are approximately 20 times higher than what we documented for Kenya.^29^ In Kenya, the elderly are least likely to seek medical care when ill,^32^ and this may have contributed to the lower than expected rates of hospitalization in this age group. The difference between rates in Kenya and the USA could also be partially explained by approaches for case ascertainment; in Kenya, only patients meeting SARI case definition were tested for influenza, whereas in the US study,^29^ influenza testing was driven by clinicians’ suspicion of influenza, allowing for less typical non‐respiratory influenza presentation to be detected. Similarly, ARI and pneumonia definitions used in the HDSS to identify SARI cases could have missed influenza‐associated cases if signs/symptoms were associated with pre‐existing underlying conditions or resulted in atypical presentations of influenza.^33^, ^34^

We also found that children 5‐14 years of age had high rates of influenza‐associated hospitalization (9‐10 per 100 000 persons) among those ≥5 years of age, similar to rates among the elderly (9‐11 per 100 000 persons). Similarly in India, children 5‐14 years of age had the second highest rates of influenza‐associated hospitalizations among individuals ≥5 years of age.^35^ In the India study, rates of hospitalization among school going children were 2 to 5 times higher than those observed among the elderly. While these findings may reflect differences in healthcare seeking between younger and older individuals, the results also suggest that school going children may be an important risk group to consider for influenza prevention strategies.

Our study had a few limitations. During our study period, patients could have sought care in facilities other than SCRH, leading to an underestimation of our SARI rates. The calculation of non‐hospitalized SARI rates is based on a healthcare utilization survey conducted >10 years ago that described the proportion of individuals aged <5 years and ≥5 years of age who were hospitalized with pneumonia. It is important to note that healthcare utilization may have changed over the past 10 years and healthcare‐seeking behavior may vary with age, especially among the elderly, which could impact the precision of non‐hospitalized case estimation. It may also be argued that hospitalized influenza‐associated SARI is in fact a more severe form of respiratory illness than non‐hospitalized influenza‐associated SARI, because individuals with more severe disease are more likely to seek medical care.^22^ The hospitalized and non‐hospitalized SARI case definitions used in the study would therefore not refer to illnesses of equal severity. In spite of these limitations, we were able to provide reasonable estimates of rates of hospitalized and non‐hospitalized influenza‐associated SARI, enabling appreciation of influenza as an important public health matter in Kenya.

## 5 CONCLUSION

Influenza virus is associated with a substantial amount of severe respiratory disease burden in Kenya, especially among very young children. Children <2 years of age could be considered as a priority group for influenza vaccination in Kenya. As influenza vaccine is not licensed for children <6 months of age, maternal vaccination of pregnant women could be considered to protect this age group.

## DISCLAIMER

The statements made and views expressed are solely the responsibility of the authors and do not necessarily represent the views of the Kenya Medical Research Institute, the African Population and Health Research Center, the University of the Witwatersrand, Wellcome Trust (UK), the Carnegie Corporation of New York, the Swedish International Development Corporation Agency, or the Centers for Disease Control and Prevention.
