# Supplementary material for: National burden of hospitalized and non‐hospitalized influenza‐associated severe acute respiratory illness in Kenya, 2012‐2014
Source: Influenza Other Respir Viruses. 2017 Dec 15;12(1):30–7. doi: 10.1111/irv.12488 (PMC5818348; doi:10.1111/irv.12488)
Supplement: Supplementary file 1 [file IRV-12-30-s001.docx]

**S1 Table:** Prevalence and 95% confidence interval limits for regional risk factors for severe acute respiratory illness and healthcare seeking behavior for acute respiratory illness in Kenya

| **Region** | **Malnutrition** | **Low birth weight** | **Non-exclusive breastfeeding** | **Household air pollution** | **Household crowding** | **HIV prevalence (children)*** | **HIV prevalence (adults)** | **Health care seeking** |
| --- | --- | --- | --- | --- | --- | --- | --- | --- |
| **Central** | 0.052  (0.041-0.067) | 0.092  (0.067-0.125) | 0.218  (0.098-0.417) | 0.768  (0.762-0.773) | 0.230  (0.214-0.246) | 0.0065 | 0.038  (0027-0.049) | 0.703  (0.595-0.792) |
| **Coast** | 0.136  (0.119-0.155) | 0.127  (0.099-0.162) | 0.262  (0.158-0.401) | 0.685  (0.679-0.691) | 0.355  (0.339-0.372) | 0.0094 | 0.043  (0.030-0.056) | 0.661  (0.572-0.740) |
| **Eastern** | 0.121  (0.108-0.137) | 0.084  (0.063-0.112) | 0.352  (0.237-0.487) | 0.833  (0.828-0.837) | 0.378  (0.363-0.394) | 0.0063 | 0.0378  (0.023-0.051) | 0.677  (0.606-0.740) |
| **Nairobi** | 0.038  (0.023-0.062) | 0.089  (0.058-0.136) | 0.131  (0.031-0.410) | 0.457  (0.448-0.465) | 0.175  (0.155-0.198) | 0.0122 | 0.049  (0.037-0.061) | 0.652  (0.459-0.806) |
| **North Eastern** | 0.190  (0.166-0.216) | 0.079  (0.046-0.133) | 0.416  (0.263-0.588) | 0.857  (0.853-0.861) | 0.666  (0.640-0.691) | 0.0019 | 0.008  (0.000-0.020) | 0.370  (0.241-0.520) |
| **Nyanza** | 0.075  (0.065-0.086) | 0.035  (0.024-0.050) | 0.340  (0.214-0.494) | 0.823  (0.819-0.828) | 0.468  (0.452-0.483) | 0.0262 | 0.151  (0.114-0.188) | 0.711  (0.644-0.769) |
| **Rift Valley** | 0.153  (0.142-0.165) | 0.066  (0.054-0.081) | 0.291  (0.205-0.395) | 0.807  (0.802-0.812) | 0.396  (0.385-0.407) | 0.0081 | 0.037  (0.024-0.050) | 0.689  (0.642-0.733) |
| **Western** | 0.091  (0.076-0.107) | 0.048  (0.031-0.074) | 0.245  (0.131-0.411) | 0.864  (0.860-0.868) | 0.488  (0.468-0.508) | 0.0086 | 0.047  (0.030-0.065) | 0.568  (0.494-0.639) |

*95% confidence interval limits were not available for the prevalence of HIV in children

Data on the prevalence of these risk factors was obtained from the Kenya Demographic Health Survey 2014 report (16) and dataset (17), the Kenya population census 2009 (18), the Kenya HIV estimates 2014 (37), and the Kenya AIDS Indicator Survey 2012 (15)
