# Supplementary material for: National burden of hospitalized and non‐hospitalized influenza‐associated severe acute respiratory illness in Kenya, 2012‐2014
Source: Influenza Other Respir Viruses. 2017 Dec 15;12(1):30–7. doi: 10.1111/irv.12488 (PMC5818348; doi:10.1111/irv.12488)
Supplement: Supplementary file 2 [file IRV-12-30-s002.docx]

**S2 Table:** Annual regional rate of hospitalized severe acute respiratory illness (SARI) in Kenya by region, 2012 to 2014

| **Year** | **Central**  **n(95% CI)** | **Coast**  **n(95% CI)** | **Eastern**  **n(95% CI)** | **Nairobi**  **n(95% CI)** | **North Eastern**  **n(95% CI)** | **Nyanza**  **n(95% CI)** | **Rift Valley**  **n(95% CI)** | **Western**  **n(95% CI)** | **Kenya**  **n(95% CI)** |
| --- | --- | --- | --- | --- | --- | --- | --- | --- | --- |
| **2012** |  |  |  |  |  |  |  |  |  |
| **<5 years** | **1,345.7**  **(1,205.2-1,510.5)** | **1,522.7**  **(1,380.9-1,680.1)** | **1,864.5**  **(1,709.0-2,021.9)** | **625.5**  **(500.1-748.8)** | **1,073.5**  **(831.0-1,387.5)** | **2,155.3**  **(1,970.4-2,341.0)** | **1,814.2**  **(1,706.4-1,925.9)** | **1,482.8**  **(1,339.9-1,649.2)** | **1,644.1**  **(1,498.4-1,801.7)** |
| **<2 years** | 2,116.2  (1,895.1-2,375.5) | 2,370.5  (2,149.7-2,615.7) | 2,949.7  (2,703.9-3,198.8) | 950.5  (760.0-1,137.8) | 1,929.0  (1,494.7-2,493.0) | 3,385.0  (3,094.7-3,676.7) | 2,872.9  (2,702.3-3,050.0) | 2,325.4  (2,101.5-2,586.4) | 2,600.3  (2,372.4-2,845.9) |
| **0-5 months** | 1,814.6  (1,625.2-2,037.7) | 2,034.7  (1,846.1-2,245.9) | 2,527.6  (2,316.7-2,740.8) | 817.9  (654.7-979.2) | 1,654.9  (1,283.9-2,138.5) | 2,904.5  (2,655.9-3,155.0) | 2,467.6  (2,320.9-2,619.6) | 1,993.6  (1,802.0-2,217.5) | 2,229.1  (2,034.3-2,439.2) |
| **6-11 months** | 2,260.7  (2,024.8-2,538.0) | 2,533.9  (2,297.9-2,795.2) | 3,147.6  (2,885.8-3,413.9) | 1,017.8  (813.1-1,218.6) | 2,059.7  (1,597.0-2,661.6) | 3,618.7  (3,308.4-3,930.8) | 3,074.2  (2,891.7-3,263.8) | 2,482.7  (2,244.0-2,761.5) | 2,776.5  (2,533.7-3,038.1) |
| **0-11 months** | 2,039.6  (1,826.8-2,290.1) | 2,286.5  (2,074.0-2,523.0) | 2,840.4  (2,603.8-3,080.4) | 918.7  (734.6-1,100.0) | 1,859.1  (1,441.9-2,402.4) | 3,264.8  (2,985.0-3,546.3) | 2,773.6  (2,608.8-2,944.6) | 2,240.4  (2,025.0-2,491.9) | 2,505.2  (2,286.3-2,741.3) |
| **12-23 months** | 2,201.3  (1,971.1-2,470.6) | 2,467.4  (2,237.1-2,722.6) | 3,066.5  (2,810.8-3,325.3) | 990.7  (792.1-1,185.6) | 2,007.2  (1,553.9-2,594.5) | 3,524.4  (3,221.9-3,827.9) | 2,994.3  (2816.5-3,178.8) | 2,418.1  (2,185.0-2,689.5) | 2,710.1  (2,471.8-2,966.7) |
| **2-4 years** | 852.4  (763.4-956.6) | 955.4  (866.3-1,053.9) | 1,187.1  (1,088.1-1,287.2) | 383.8  (306.9-459.5) | 7,76.3  (600.4-1,003.4) | 1,364.7  (1,247.6-1,482.2) | 1,159.3  (1,090.4-1,230.7) | 936.4  (846.0-1,041.4) | 1,048.3  (953.9-1,151.1) |
| **≥5 years** | **49.4**  **(44.6-53.4)** | **47.3**  **(43.0-51.3)** | **61.5**  **(57.5-65.1)** | **18.9**  **(15.2-22.0)** | **36.0**  **(27.9-46.6)** | **112.0**  **(101.6-122.1)** | **60.7**  **(57.8-63.5)** | **61.1**  **(56.0-66.0)** | **60.4**  **(55.4-65.3)** |
| **5-14 years** | 76.7  (69.2-82.9) | 72.2  (65.7-78.2) | 84.8  (79.5-89.6) | 48.5  (38.8-56.4) | 50.5  (39.1-65.3) | 105.1  (98.5-110.7) | 86.2  (82.6-89.7) | 77.9  (71.3-84.0) | 80.8(  74.6-86.7) |
| **15-49 years** | 33.7  (30.5-36.5) | 32.4  (29.4-35.1) | 43.1  (40.3-45.7) | 10.9  (8.7-12.7) | 22.6  (17.5-29.2) | 101.6  (90.6-112.4) | 42.4  (40.2-44.5) | 45.1  (41.3-48.7) | 44.1  (40.2-47.9) |
| **50-64 years** | 39.7  (36.0-43.1) | 38.2  (34.8-41.5) | 50.6  (47.4-53.8) | 13.4  (11.3-16.2) | 26.6  (21.3-34.6) | 119.3  (106.5-132.0) | 49.8  (47.3-52.4) | 53.0  (48.5-57.5) | 54.7  (50.0-59.5) |
| **65+ years** | 94.0  (85.3-101.9) | 90.2  (82.0-98.3) | 120.2  (112.4-127.6) | 31.4  (26.2-36.7) | 63.3  (49.9-82.5) | 282.9  (252.4-313.4) | 118.0  (112.0-124.0) | 125.9  (115.7-136.2) | 133.4  (122.0-144.7) |
| **All ages** | **207.9**  **(186.5-231.5)** | **286.1**  **(259.5-314.9)** | **319.1**  **(293.5-344.7)** | **95.7**  **(76.6-114.1)** | **183.3**  **(142.0-237.0)** | **468.0**  **(427.2-508.7)** | **345.7**  **(325.8-366.2)** | **309.7**  **(280.5-342.9)** | **304.2**  **(277.5-332.6)** |
|  |  |  |  |  |  |  |  |  |  |
| **2013** |  |  |  |  |  |  |  |  |  |
| **<5 years** | **967.7**  **(866.9-1,086.2)** | **1,089.3**  **(987.9-1,201.9)** | **1,344.7**  **(1,232.7-1,458.2)** | **442.4**  **(353.8-529.7)** | **822.0**  **(636.3-1062.5)** | **1,548.1**  **(1,415.3-1,681.4)** | **1,307.6**  **(1,229.9-1,388.1)** | **10,65.0**  **(962.5-1,184.5)** | **1,185.0**  **(1,079.7-1,299.0)** |
| **<2 years** | 1,267.0  (1,135.5-1,422.5) | 1,417.4  (1,285.8-1,564.1) | 1,767.3  (1,620.3-1,916.6) | 567.7  (454.3-680.1) | 1,153.7  (893.4-1,491.5) | 2,023.6  (1,850.1-2,197.9) | 1,715.2  (1,613.4-1,820.9) | 1,392.6  (1,258.7-15,49.1) | 1,554.9  (1,418.8-1,701.9) |
| **0-5 months** | 517.1  (464.4-581.2) | 577.7  (524.4-637.4) | 718.8  (659.0-779.8) | 233.6  (187.6-279.5) | 470.8  (365.3-608.8) | 825.6  (755.5-897.5) | 701.1  (659.7-744.6) | 566.6  (513.0-630.7) | 633.7  (578.8-693.8) |
| **6-11 months** | 1,790.5  (1,604.5-2,010.4) | 2,007.7  (1,821.2-2,215.9) | 2,494.0  (2,286.7-2,704.6) | 806.7  (645.0-966.6) | 1,630.7  (1263.9-2,109.2) | 2,866.4  (2,620.5-3,113.1) | 2,435.1  (2,290.7-2,585.2) | 1,967.4  (1,778.5-2,188.4) | 2,199.5  (2,007.5-2,406.9) |
| **0-11 months** | 1,159.5  (1,039.5-1,302.2) | 1,299.1  (1,178.6-1,433.7) | 1,614.4  (1,480.2-1,750.8) | 522.7  (418.4-626.1) | 1,056.0  (818.6-1,365.7) | 1,855.2  (1,696.4-2,015.2) | 1,575.9  (1,482.5-1,673.2) | 1,273.3  (1,151.4-1,416.6) | 1,423.6  (1,299.5-1,558.0) |
| **12-23 months** | 1,386.6  (1,242.3-1,556.3) | 1,553.8  (1,409.4-1,714.4) | 1,930.7  (1,770.0-2,093.7) | 624.7  (499.8-748.4) | 1,263.1  (977.2-1,632.4) | 2,219.0  (2,028.5-2,409.9) | 1,885.5  (1,773.3-2,001.5) | 1,522.8  (1,375.9-1,693.7) | 1,706.5  (1,556.6-1,868.1) |
| **2-4 years** | 776.0  (694.9-870.9) | 869.7  (788.6-959.5) | 1,080.9  (990.8-1,172.1) | 349.3  (279.1-417.9) | 706.8  (547.0-913.5) | 1,242.4  (1,135.7-1,349.3) | 10,55.5  (992.8-1,120.4) | 852.5  (770.4-948.1) | 954.4  (868.4-1,047.9) |
| **≥5 years** | **36.6**  **(33.1-39.6)** | **36.6**  **(33.3-39.7)** | **46.1**  **(43.2-48.8)** | **15.1**  **(12.2-17.6)** | **30.3**  **(23.5-39.3)** | **78.1**  **(71.4-84.5)** | **47.3**  **(45.1-49.4)** | **46.5**  **(42.6-50.3)** | **45.4**  **(41.7-49.0)** |
| **5-14 years** | 78.3  (70.7-84.6) | 73.7  (67.0-79.8) | 86.5  (81.2-91.4) | 49.4  (39.6-57.6) | 51.5  (39.9-66.7) | 107.3  (100.5-113.0) | 88.0  (84.3-91.6) | 79.5  (72.7-85.7) | 82.5  (76.1-88.5) |
| **15-49 years** | 18.5  (16.7-20.0) | 17.7  (16.1-19.1) | 23.6  (22.0-25.0) | 6.0  (4.8-6.9) | 12.4  (9.6-16.0) | 55.5  (49.5-61.4) | 23.2  (21.9-24.3) | 24.6  (22.5-26.7) | 24.1  (21.9-26.2) |
| **50-64 years** | 38.9  (35.4-42.2) | 37.2  (33.9-40.4) | 49.5  (46.4-52.6) | 13.0  (11.0-15.8) | 26.8(  20.7-35.4) | 116.7  (104.3-129.4) | 48.7  (46.2-51.2) | 52.0  (47.6-56.3) | 53.5  (48.9-58.3) |
| **65+ years** | 21.1  (19.0-23.1) | 20.4  (18.6-22.2) | 26.8  (25.2-28.4) | 7.7  (7.7-10.2) | 14.9  (13.1-20.5) | 63.0  (56.7-69.9) | 26.4  (25.2-28.0) | 28.3  (26.1-31.1) | 29.9  (27.5-32.6) |
| **All ages** | **150.4**  **(135.0-167.5)** | **207.0**  **(187.8-227.7)** | **231.7**  **(213.2-250.2)** | **69.3**  **(55.5-82.5)** | **142.8**  **(110.6-184.6)** | **334.2**  **(305.5-362.7)** | **252.1**  **(237.7-267.0)** | **224.6**  **(203.5-248.6)** | **220.8**  **(201.4-241.4)** |
|  |  |  |  |  |  |  |  |  |  |
| **2014** |  |  |  |  |  |  |  |  |  |
| **<5 years** | **604.5**  **(541.7-678.7)** | **682.2**  **(618.8-752.9)** | **838.2**  **(768.5-909.0)** | **278.9**  **(223.2-334.0)** | **502.3**  **(389.2-649.7)** | **968.1**  **(885.0-1,051.4)** | **817.6**  **(769.0-868.0)** | **665.0**  **(601.0-739.7)** | **740.0**  **(674.4-811.2)** |
| **<2 years** | 447.1  (400.5-501.7) | 500.9  (454.3-552.8) | 622.6  (570.7-675.1) | 201.3  (160.8-241.1) | 407.4  (315.3-526.5) | 1,360.9  (1,244.3-1,478.2) | 607.9  (571.7-645.3) | 490.9  (443.7-546.0) | 549.7  (500.2-603.6) |
| **0-5 months** | 902.3  (808.8-1013.4) | 1010.8  (917.1-1,115.2) | 1,256.5  (1,151.6-1,362.5) | 406.5  (325.9-487.0) | 822.1  (639.4-1,064.4) | 1,443.7  (1,319.9-1,568.4) | 1,226.1  (1,153.4-1,301.9) | 990.2  (895.5-1,102.0) | 1,107.7  (1,011.2-1,212.4) |
| **6-11 months** | 819.8  (734.3-921.0) | 918.9  (834.4-1,013.9) | 1,141.0  (1,046.6-1,237.5) | 369.8  (296.2-443.4) | 749.0  (581.3-971.4) | 1,311.4  (1,199.4-1,424.2) | 1,114.0  (1,048.2-1,182.9) | 899.8  (813.5-1,000.6) | 1,006.5  (918.9-1,101.6) |
| **0-11 months** | 860.7  (771.2-966.8) | 964.4  (875.4-1,064.1) | 1,198.2  (1,098.6-1,299.4) | 388.0  (311.0-465.0) | 785.2  (610.1-1,017.5) | 1,377.0  (1,259.1-1,495.6) | 1,169.6  (1,100.3-1,241.9) | 944.6  (854.1-1,050.9) | 1,056.7  (964.7-1,156.5) |
| **12-23 months** | 839.1  (752.1-942.1) | 940.1  (852.4-1,037.6) | 1,168.0  (1,071.1-1,266.8) | 377.3  (302.1-451.4) | 764.5  (592.6-989.2) | 1,342.3  (1,227.1-1,458.0) | 1,140.6  (1,073.0-1,211.0) | 921.2  (832.3-1,024.8) | 1,032.4  (941.8-1,130.3) |
| **2-4 years** | 850.4  (762.2-955.1) | 953.1  (864.7-1,051.8) | 1,183.6  (1,085.3-1,283.6) | 383.3  (307.0-459.0) | 775.5  (601.9-1,004.1) | 715.5  (654.0-777.0) | 1,156.5  (1,088.0-1,228.0) | 933.4  (843.7-1,038.4) | 1,045.4  (954.1-1,144.4) |
| **≥5 years** | **37.5**  **(33.9-40.6)** | **36.0**  **(32.8-39.0)** | **46.5**  **(43.5-49.3)** | **14.4**  **(11.6-16.9)** | **27.1**  **(21.0-35.1)** | **86.5**  **(78.3-94.4)** | **46.0**  **(43.8-48.1)** | **46.4**  **(42.5-50.2)** | **46.0**  **(42.2-49.8)** |
| **5-14 years** | 54.3  (49.1-58.7) | 51.1  (46.5-55.4) | 60.0  (56.3-63.5) | 34.4  (27.6-40.1) | 35.8  (27.8-46.4) | 74.5  (69.8-78.5) | 61.1  (58.5-63.6) | 55.2  (50.5-59.6) | 57.2  (52.8-61.5) |
| **15-49 years** | 27.3  (24.7-29.5) | 26.2  (23.8-28.4) | 34.9  (32.6-37.0) | 8.8  (7.1-10.3) | 18.4  (14.2-23.7) | 82.2  (73.3-91.0) | 34.3  (32.5-36.1) | 36.5  (33.4-39.4) | 35.7  (32.5-38.8) |
| **50-64 years** | 47.5  (42.9-51.5) | 45.4  (41.4-49.5) | 60.6  (56.7-64.2) | 15.4  (12.7-18.1) | 32.1  (25.3-42.2) | 142.5  (127.1-157.9) | 59.6  (56.5-62.7) | 63.4  (58.1-68.7) | 65.4  (59.7-71.1) |
| **65+ years** | 47.2  (42.8-51.1) | 45.1  (41.7-49.5) | 59.8  (56.0-63.6) | 17.5  (15.0-22.5) | 32.9  (25.6-43.9) | 141.3  (126.1-156.5) | 58.9  (55.9-61.9) | 63.0  (58.1-68.4) | 66.7  (61.1-72.6) |
| **All ages** | **106.8**  **(96.0-118.6)** | **140.6**  **(127.6-154.5)** | **159.6**  **(147.1-172.1)** | **48.0**  **(38.4-57.1)** | **94.5**  **(73.3-122.4)** | **240.1**  **(218.9-261.1)** | **171.4**  **(161.7-181.4)** | **154.6**  **(140.1-170.7)** | **152.8**  **(139.5-167.0)** |
|  |  |  |  |  |  |  |  |  |  |
| **2012-2014** |  |  |  |  |  |  |  |  |  |
| **<5 years** | **982.3**  **(879.7-1102.6)** | **1,109.3**  **(1,005.9-1,224.0)** | **1,362.6**  **(1,248.9-1,477.6)** | **453.2**  **(362.7-542.7)** | **806.8**  **(624.3-1043.2)** | 1,572.9  (1,437.9-1,708.4) | **1,326.5**  **(1,247.7-1,408.1)** | **1,081.6**  **(977.5-1,203.0)** | **1,201.6**  **(1,095.0-1,317.1)** |
| **<2 years** | 1,427.1  (1,278.3-1,601.8) | 1,598.4  (1,449.5-1,764.1) | 1,989.0  (1,823.0-2,157.0) | 640.7  (512.8-767.6) | 1300.0  (1006.4-1682.0) | 2,282.4  (2,086.6-2,479.0) | 1,937.1  (1,822.2-2,056.4) | 1,567.9  (1,417.2-1,743.9) | 1,753.3  (1,599.7-1,919.0) |
| **0-5 months** | 1,090.0  (976.2-1,223.5) | 1,221.6  (1,108.4-1,348.9) | 1,517.7  (1,391.1-1,646.5) | 490.8  (393.0-588.6) | 991.8  (768.2-1284.4) | 1,743.8  (1,594.3-1,894.1) | 1,481.3  (1,393.6-1,572.7) | 1,196.7  (1,082.3-1,331.0) | 1,338.2  (1,221.4-1,464.6) |
| **6-11 months** | 1,652.1  (1,480.4-1,854.5) | 1,851.5  (1,678.6-2,043.0) | 2,299.9  (2,107.9-2,494.2) | 743.9  (595.1-890.7) | 1505.3  (1165.9-1948.5) | 2,644.0  (2,417.5-2,872.2) | 2,246.4  (2,113.3-2,384.8) | 1,814.3  (1,639.9-2,018.4) | 2,028.9  (1,851.5-2,220.2) |
| **0-11 months** | 1,373.6  (1,230.6-1,541.9) | 1,539.4  (1,396.0-1,699.1) | 1,912.3  (1,752.7-2,074.1) | 618.5  (495.0-741.0) | 1250.8  (968.8-1619.4) | 2,197.9  (2,009.6-2,387.6) | 1,867.3  (1,756.7-1,982.4) | 1,508.3  (1,363.6-1,677.8) | 1,686.6  (1,539.3-1,845.8) |
| **12-23 months** | 1,486.7(  1,331.3-1,668.4) | 1,666.6  (1,511.2-1,839.1) | 2,071.0  (1,898.1-2,245.6) | 668.9  (535.4-801.2) | 1355.1  (1048.5-1752.0) | 2,380.4  (2,176.0-2,585.2) | 2,022.3  (1,902.3-2,146.9) | 1,633.0  (1,475.8-1,816.1) | 1,830.3  (1,669.4-2,003.6) |
| **2-4 years** | 697.5  (624.5-782.9) | 781.9  (709.0-862.6) | 971.6  (890.6-1,053.6) | 313.8  (251.1-375.4) | 635.4  (491.6-821.3) | 1,116.7  (1,020.8-1,212.9) | 948.8  (892.4-1,007.2) | 766.2  (692.4-852.2) | 857.9  (780.6-942.0) |
| **≥5 years** | **41.5**  **(37.5-44.9)** | **40.3**  **(36.7-43.7)** | **51.8**  **(48.5-54.9)** | **16.3**  **(13.0-19.0)** | **31.3**  **(24.3-40.5)** | **93.0**  **(84.5-101.1)** | **51.7**  **(49.3-54.1)** | **51.8**  **(47.4-56.0)** | **51.0**  **(46.8-55.1)** |
| **5-14 years** | 70.4  (63.6-76.1) | 66.3  (60.3-71.8) | 77.9  (73.0-82.3) | 44.5  (35.6-51.8) | 46.3  (35.8-59.9) | 96.6  (90.5-101.7) | 79.1  (75.8-82.4) | 71.5  (65.4-77.1) | 74.2  (68.5-79.7) |
| **15-49 years** | 26.4  (23.9-28.6) | 25.4  (23.1-27.5) | 33.7  (31.5-35.8) | 8.6  (6.9-10.0) | 17.8  (13.7-23.0) | 79.5  (70.9-88.0) | 33.2  (31.5-34.9) | 35.3  (32.3-38.2) | 34.5  (31.4-37.5) |
| **50-64 years** | 43.7  (39.5-47.2) | 41.9  (38.1-45.6) | 55.8  (52.2-59.1) | 14.4  (11.7-17.2) | 29.4  (23.3-38.1) | 131.2  (117.1-145.3) | 54.8  (52.0-57.6) | 58.4  (53.7-63.2) | 60.2  (55.0-65.4) |
| **65+ years** | 55.6  (50.3-60.0) | 53.3  (48.8-57.7) | 71.0(66.4-75.3) | 20.4  (17.9-25.6) | 37.4  (29.9-48.6) | 167.5  (149.6-185.3) | 69.9  (66.2-73.5) | 74.5  (68.4-80.6) | 78.9  (72.3-85.6) |
| **All ages** | **156.5**  **(140.5-174.2)** | **213.3**  **(193.5-234.7)** | **239.2**  **(220.1-258.2)** | **71.6**  **(57.3-85.3)** | **141.5**  **(109.5-182.9)** | **350.8**  **(320.3-381.2)** | **258.9**  **(244.1-274.2)** | **231.9**  **(210.1-256.5)** | **228.1**  **(208.1-249.4)** |

*Rate per 100,000 persons
