# Supplementary material for: National burden of hospitalized and non‐hospitalized influenza‐associated severe acute respiratory illness in Kenya, 2012‐2014
Source: Influenza Other Respir Viruses. 2017 Dec 15;12(1):30–7. doi: 10.1111/irv.12488 (PMC5818348; doi:10.1111/irv.12488)
Supplement: Supplementary file 3 [file IRV-12-30-s003.docx]

**S3 Table:** Annual regional rate of non-hospitalized severe acute respiratory illness (SARI) in Kenya by region, 2012 to 2014

| **Year** | **Central**  **n(95% CI)** | **Coast**  **n(95% CI)** | **Eastern**  **n(95% CI)** | **Nairobi**  **n(95% CI)** | **North Eastern**  **n(95% CI)** | **Nyanza**  **n(95% CI)** | **Rift Valley**  **n(95% CI)** | **Western**  **n(95% CI)** | **Kenya**  **n(95% CI)** |
| --- | --- | --- | --- | --- | --- | --- | --- | --- | --- |
| **2012** |  |  |  |  |  |  |  |  |  |
| **<5 years** | **3,882.1**  **(3,476.5-4,357.3)** | **4,778.1**  **(4,332.9-5,271.6)** | **5,667.4**  **(5,195.0-6,145.9)** | **1,994.9**  **(1,594.8-2,387.5)** | **7,208.2**  **(5,579.9-9,316.4)** | **6,134.5**  **(5,608.3-6,663.0)** | **5,460.7**  **(5,136.2-5,797.1)** | **5,684.1**  **(5,136.3-6,322.0)** | **5,285.3**  **(4,784.7-5,838.5)** |
| **<2 years** | 6,105.0  (5,466.9-6,852.9) | 7,438.5  (6,745.3-8,207.4) | 8,966.3  (8,219.3-9,723.5) | 3,031.7  (2,423.6-3,627.8) | 12,952.9  (10,036.5-16,741.0) | 9,634.6  (8,808.2-10,464.8) | 8,647.7  (8,133.9-9,180.4) | 8,914.1  (8,055.8-9,914.8) | 8,281.2  (7,515.2-9,121.6) |
| **0-5 months** | 5,235.7  (4,687.7-5,878.3) | 6,384.7  (5,793.0-7,047.4) | 7,683.7  (7,042.8-8,331.5) | 2,609.1  (2,088.1-3,122.3) | 11,113.4  (8,620.6-14,360.7) | 8,267.7  (7,559.1-8,980.0) | 7,427.2  (6,986.2-7,885.1) | 7,642.8  (6,907.6-8,501.3) | 7,090.7  (6,437.3-7,808.3) |
| **6-11 months** | 6,521.4  (5,842.0-7,322.0) | 7,951.7  (7,210.5-8,770.4) | 9,568.0  (8,772.3-10,377.4) | 3,246.4  (2,593.7-3,885.7) | 13,832.4  (10,724.4-17,874.0) | 10,299.7  (9,416.4-11,187.6) | 9,253.7  (8,703.9-9,823.9) | 9,517.0  (8,602.0-10,586.0) | 8,831.6  (8,017.3-9,725.0) |
| **0-11 months** | 5,884.3  (5270.0-6,606.6) | 7,175.2  (6,508.1-7,916.6) | 8,634.3  (7,915.3-9,363.6) | 2,930.6  (2,343.1-3,507.4) | 12,485.0  (9,681.9-16,133.0) | 9,292.8  (8,496.0-10,093.7) | 8,348.6  (7,852.7-8,863.2) | 8,588.3  (7,762.4-9,553.0) | 7,968.9  (7,234.4-8,775.2) |
| **12-23 months** | 6,350.4  (5,685.9-7,126.7) | 7,742.1  (7,018.9-8,542.8) | 9,321.0  (8,543.9-10,107.8) | 3,159.7  (2,525.5-3,780.2) | 13,476.7  (1,0433.6-17,421.7) | 10,031.1  (9,170.3-10,895.2) | 9,013.0  (8,477.4-9,568.0) | 9,269.7  (8,376.0-10,309.7) | 8,641.8  (7,839.5-9,521.6) |
| **2-4 years** | 2,458.8  (2,202.0-2,759.4) | 2,997.5  (2,718.3-3,306.8) | 3,608.4  (3,307.4-3,912.9) | 1,223.9  (978.5-1,465.1) | 5,212.4  (4,031.6-6,736.9) | 3,884.1  (3,551.0-4,218.6) | 3,489.5  (3,282.0-3,704.3) | 3,589.4  (3,243.0-3,992.0) | 3,418.6  (3,083.3-3,792.9) |
| **≥5 years** | **262.2**  **(237.1-283.6)** | **270.9**  **(246.3-293.5)** | **342.0**  **(320.2-362.1)** | **109.8**  **(88.0-128.0)** | **415.3**  **(322.3-537.3)** | **588.2**  **(533.4-640.8)** | **334.7**  **(318.9-350.1)** | **419.0**  **(383.6-452.6)** | **351.9**  **(320.7-383.2)** |
| **5-14 years** | 407.3  (367.8-440.2) | 413.5  (375.8-447.5) | 471.7  (442.4-498.4) | 281.4  (225.3-327.8) | 582.2  (450.8-753.0) | 552.1  (517.4-581.4) | 475.4  (455.4-495.0) | 533.8  (488.6-575.7) | 479.9  (439.1-520.9) |
| **15-49 years** | 179.2  (162.0-193.8) | 185.4  (168.5-200.7) | 239.8  (224.1-254.1) | 63.4  (50.7-73.8) | 260.9  (202.3-337.2) | 533.3  (475.8-590.2) | 233.9  (221.5-245.7) | 309.0  (282.8-334.1) | 253.4  (229.8-276.7) |
| **50-64 years** | 211.2  (191.5-228.9) | 218.5  (199.4-237.6) | 281.7  (264.0-299.4) | 78.1  (65.5-94.3) | 307.8  (245.7-399.1) | 626.4  (559.5-693.2) | 275.0  (261.0-289.0) | 363.4  (332.6-394.3) | 313.2  (285.6-342.2) |
| **65+ years** | 499.6  (453.4-541.6) | 516.4  (470.0-562.9) | 669.0  (626.0-709.9) | 183.4  (154.6-214.9) | 730.9  (575.6-953.5) | 1,485.5  (1,325.5-1,646.0) | 651.4  (618.2-684.2) | 863.4  (793.3-934.1) | 758.9  (693.0-826.2) |
| **All ages** | **704.8**  **(633.2-781.7)** | **1,000.3**  **(907.6-1,099.1)** | **1,103.0**  **(1,016.8-1,188.6)** | **348.6**  **(279.0-414.3)** | **1,380.0**  **(1,069.0-1784.0)** | **1,554.5**  **(1,417.6-1,690.1)** | **1,167.9**  **(1,101.9-1235.4)** | **1,339.7**  **(1,214.7-1,479.0)** | **1,111.2**  **(1,007.8-1,222.8)** |
|  |  |  |  |  |  |  |  |  |  |
| **2013** |  |  |  |  |  |  |  |  |  |
| **<5 years** | **2,791.7**  **(2,500.9-3,133.5)** | **3,418.1**  **(3,100.1-3,771.3)** | **4,087.7**  **(3,747.2-4,432.6)** | **1,410.7**  **(1,128.6-1,689.2)** | **5,519.8**  **(4,272.9-7,134.5)** | **4,406.2**  **(4,028.4-4,785.9)** | **3,935.9**  **(3,702.1-4,178.2)** | **4,082.5**  **(3,689.6-4,540.9)** | **3,818.8**  **(3,455.2-4,221.8)** |
| **<2 years** | 3,655.4  (3,276.0-4,103.9) | 4,448.1  (4,034.8-4,907.7) | 5,372.7  (4,925.7-5,826.1) | 1,810.2  (14,49.2-2,169.1) | 7,747.7  (6,000.1-10,015.8) | 5,759.7  (5,266.3-6,256.3) | 5,163.0  (4,856.4-5,481.0) | 5,338.6  (4,825.4-5,938.5) | 4,952.4  (4,495.0-5,455.5) |
| **0-5 months** | 1,491.9  (1,340.4-1,677.9) | 1,813.3  (1,645.3-2,000.1) | 2,185.8  (2,004.2-2,370.9) | 744.7  (599.2-892.1) | 3,161.9  (2,455.7-4,091.4) | 2,350.3  (2,150.9-2,555.1) | 2,110.8  (1,986.1-2,241.3) | 2,172.0  (1,966.8-2,418.0) | 2,016.1  (1,832.2-2,221.5) |
| **6-11 months** | 5,166.0  (4,629.0-5,800.1) | 6,300.8  (5,714.8-6,953.1) | 7,581.7  (6,951.0-8,221.3) | 2,572.5  (2,057.2-3,082.1) | 10,952.5  (8,488.5-14,162.1) | 8,158.5  (7,459.3-8,861.2) | 7,329.7  (6,894.9-7,781.7) | 7,542.1  (6,817.2-8,389.5) | 6,996.5  (6,352.0-7,704.9) |
| **0-11 months** | 3,345.4  (2,999.4-3,757.5) | 4,077.1  (3,698.3-4,498.8) | 4,907.9  (4,499.7-5,322.3) | 1,666.8  (1,334.8-1,996.9) | 7,092.0  (5,499.0-9,171.7) | 5,280.4  (4,828.9-5,736.4) | 4,743.6  (4,462.5-5,036.3) | 4,881.1  (4,413.8-5,430.5) | 4,528.6  (4,112.3-4,987.8) |
| **12-23 months** | 4,000.1  (3,583.6-4,489.2) | 4,875.9  (4,422.9-5,379.2) | 5,869.0  (5,380.5-6,364.1) | 1,991.8  (1,594.2-2,387.0) | 8,481.8  (6,561.1-10,960.8) | 6,315.7  (5,773.7-68,59.3) | 5,675.4  (5,337.7-6,024.3) | 5,837.8  (5,274.7-6,493.0) | 5,441.8  (4,936.9-5,995.6) |
| **2-4 years** | 2,238.7  (2,004.6-2,512.2) | 2,728.8  (2,474.5-3,010.7) | 3,285.7  (3,011.7-3,562.9) | 1,113.6  (890.1-1,332.4) | 4,745.7  (3,672.8-6,133.5) | 3,536.0  (3,232.4-3,840.4) | 3,176.9  (2,988.2-3,372.4) | 3,267.8  (2,953.0-3,634.5) | 3,112.4  (2,807.3-3,453.0) |
| **≥5 years** | **194.3**  **(175.7-210.2)** | **209.7**  **(190.7-227.1)** | **256.6**  **(240.5-271.6)** | **87.9**  **(70.7-102.5)** | **350.0**  **(271.5-453.5)** | **410.3**  **(374.9-443.8)** | **260.9**  **(249.1-272.7)** | **319.1**  **(292.1-344.7)** | **265.9**  **(242.6-289.4)** |
| **5-14 years** | 415.8  (375.6-449.3) | 422.1  (383.7-457.0) | 481.4  (451.6-508.7) | 287.1  (229.7-334.3) | 594.5  (460.5-769.1) | 563.4  (527.8-593.4) | 485.2  (464.9-505.3) | 544.7  (498.3-587.3) | 489.9  (448.2-531.7) |
| **15-49 years** | 98.1  (88.7-106.1) | 101.2  (92.0-109.5) | 131.1  (122.5-138.9) | 34.7  (27.9-40.3) | 143.2  (110.9-185.2) | 291.2  (259.9-322.3) | 127.8  (121.1-134.3) | 168.8  (154.5-182.7) | 138.5  (125.6-151.3) |
| **50-64 years** | 207.0  (188.1-224.6) | 212.7  (194.2-231.3) | 275.6  (258.4-292.9) | 76.1  (63.7-91.8) | 309.2  (239.2-408.5) | 612.7  (547.6-679.4) | 268.7  (255.1-282.5) | 356.3  (326.2-386.3) | 306.8  (279.4-335.7) |
| **65+ years** | 112.2  (101.3-122.7) | 117.1  (107.3-127.7) | 149.4  (140.2-158.5) | 45.9  (45.9-61.2) | 173.7  (151.3-237.2) | 331.2  (297.9-367.3) | 146.0  (139.2-154.3) | 194.2  (179.2-213.1) | 170.4  (156.9-187.0) |
| **All ages** | **511.9**  **(460.0-567.6)** | **728.9**  **(661.5-800.6)** | **804.1**  **(741.6-866.2)** | **255.5**  **(204.7-303.6)** | **1,084.2**  **(839.8-1,402.4)** | **1,106.5**  **(1,011.5-1,200.3)** | **858.3**  **(810.3-907.5)** | **977.1**  **(886.2-10,78.5)** | **812.7**  **(737.0-894.6)** |
|  |  |  |  |  |  |  |  |  |  |
| **2014** |  |  |  |  |  |  |  |  |  |
| **<5 years** | **1,744.2**  **(1,562.9-1,958.0)** | **2,140.9**  **(1,942.0-2,362.4)** | **2,548.0**  **(2,336.1-2,763.1)** | **889.7**  **(712.1-1,065.3)** | **3,372.8**  **(2,613.5-4,362.6)** | **2,755.4**  **(2,519.2-2,992.7)** | **2,460.9**  **(2,314.9-2,612.8)** | **2,549.3**  **(2,304.2-2,835.8)** | **2,382.7**  **(2,156.7-2,633.7)** |
| **<2 years** | 2,453.8  (2,199.0-2,755.6) | 2,991.1  (2,713.9-3,300.5) | 3,598.4  (3,299.4-3,902.1) | 1,222.5  (979.8-14,64.2) | 5,207.8  (4,042.5-6,743.1) | 3,873.7  (3,542.2-4,207.5) | 3,481.4  (3,275.2-3,696.5) | 3,578.4  (3,234.7-3,980.8) | 3,329.3  (3,022.7-3,668.1) |
| **0-5 months** | 2,603.7  (2,333.0-2,924.3) | 3,172.1  (2,878.7-3,499.3) | 3,820.4  (3,501.3-4,141.7) | 1,296.3  (1,039.7-1,552.9) | 5,520.5  (4,293.3-7,148.8) | 4,109.4  (3,757.6-4,463.9) | 3,690.8  (3,472.1-3,918.8) | 3,796.5  (3,433.7-4,224.3) | 3,523.8  (3,200.4-3,881.3) |
| **6-11 months** | 2,366.0  (2,119.1-2,657.2) | 2,883.4  (2,619.3-3,182.1) | 3,468.4  (3,182.1-3,762.4) | 1,179.4  (945.8-1,415.0) | 5,032.6  (3,905.1-6,522.8) | 3,732.9  (3,414.5-4,054.0) | 3,353.3  (3,155.4-3,560.9) | 3,450.1  (3,118.4-3,836.7) | 3,202.2  (2,908.5-3,527.6) |
| **0-11 months** | 2,483.8  (2,225.1-2,789.5) | 3,026.5  (2,747.9-3,339.3) | 3,642.8  (3,340.3-3,950.3) | 1,237.3  (992.3-1,483.3) | 5,274.3  (4,097.5-6,833.0) | 3,919.5  (3,584.5-4,257.1) | 3,520.5  (3,312.4-3,738.2) | 3,621.7  (3,274.7-4,028.7) | 3,361.6  (3,053.2-3,702.9) |
| **12-23 months** | 2,420.5  (2,170.1-2,717.9) | 2,950.3  (2,674.7-3,255.8) | 3,550.9  (3,255.8-3,850.6) | 1,203.7  (963.9-1,439.9) | 5,133.3  (3,981.1-6,642.4) | 3,820.7  (3,493.0-4,149.9) | 3,433.5  (3,229.8-3,645.4) | 3,531.0  (3,191.1-3,928.4) | 3,292.1  (2,987.5-3,628.0) |
| **2-4 years** | 1,289.9  (1,155.6-1,447.3) | 1,571.8  (1,425.3-1,734.5) | 1,892.4  (1,734.9-2,052.2) | 642.2  (513.0-768.7) | 2,735.3  (2,117.0-3,535.5) | 2,036.4  (1,861.6-2,211.6) | 1,829.8  (1,720.9-1,942.5) | 1,882.0  (1,700.8-2,093.2) | 1,792.8  (1,617.1-1,989.1) |
| **≥5 years** | **199.2**  **(180.1-215.5)** | **206.2**  **(187.5-223.5)** | **258.8**  **(242.3-274.1)** | **83.9**  **(67.5-98.1)** | **312.2**  **(242.7-404.9)** | **454.2**  **(411.2-495.5)** | **253.7**  **(241.7-265.5)** | **318.1**  **(291.2-343.9)** | **267.9**  **(244.1-291.9)** |
| **5-14 years** | 288.6  (260.8-311.9) | 292.8  (266.1-317.0) | 334.2  (313.4-353.3) | 199.9  (160.4-233.0) | 413.1  (320.7-534.8) | 391.2  (366.5-412.0) | 336.9  (322.9-350.9) | 378.3  (346.0-408.3) | 340.1  (311.3-369.4) |
| **15-49 years** | 145.0  (131.2-156.9) | 149.9  (136.3-162.4) | 194.1  (181.4-205.7) | 51.3  (41.2-60.0) | 211.7  (164.3-273.9) | 431.6  (385.0-477.5) | 189.4  (179.4-198.9) | 250.0  (228.6-270.4) | 205.1  (186.0-224.0) |
| **50-64 years** | 252.5  (228.1-273.7) | 260.4  (236.8-283.6) | 337.2  (315.3-357.5) | 89.8  (74.4-105.3) | 370.9  (293.2-487.6) | 748.5  (667.5-829.2) | 328.9  (311.8-346.1) | 434.4  (398.4-470.7) | 374.4  (340.7-409.1) |
| **65+ years** | 250.9  (227.5-271.9) | 258.6  (238.6-283.7) | 332.9  (311.5-354.3) | 102.4  (87.4-132.3) | 380.2  (296.1-506.3) | 742.1  (662.1-821.7) | 325.3  (308.8-341.9) | 432.2  (398.5-469.1) | 380.0  (347.2-415.0) |
| **All ages** | **388.1**  **(349.2-428.5)** | **519.2**  **(471.4-569.6)** | **586.0**  **(541.5-629.8)** | **186.0**  **(149.2-220.6)** | **746.9**  **(579.4-966.9)** | **855.1**  **(778.5-930.6)** | **612.5**  **(578.6-647.0)** | **708.3**  **(643.2-779.6)** | **593.4**  **(538.5-652.4)** |
|  |  |  |  |  |  |  |  |  |  |
| **2012-2014** |  |  |  |  |  |  |  |  |  |
| **<5 years** | **2,833.8**  **(2,537.9-3,180.7)** | **3,480.7**  **(3,156.4-3,840.6)** | **4,142.2**  **(3,796.5-4,491.5)** | **1,445.6**  **(1,156.7-1,730.8)** | **5,417.4**  **(4,192.3-7,004.5)** | **4,476.9**  **(4,092.7-4,862.6)** | **3,992.6**  **(3,755.8-4,238.5)** | **4,146.3**  **(3,747.4-4,611.5)** | **3,867.3**  **(3,500.1-4,273.7)** |
| **<2 years** | 4,117.1  (3,687.7-4,620.9) | 5,015.6  (4,548.3-5,535.1) | 6,046.6  (5,541.7-6,556.8) | 2,043.7  (1,635.5-2,448.2) | 8,731.1  (6,758.7-11,294.0) | 6,496.4  (5,939.2-7,056.3) | 5,830.6  (5,485.1-6,190.0) | 6,010.7  (5,433.1-6,685.2) | 5,583.8  (5,067.5-6,151.0) |
| **0-5 months** | 3,144.6  (2,816.5-3,530.5) | 3,832.9  (3,477.6-4,232.2) | 4,614.1  (4,228.7-5,005.1) | 1,566.4  (1,253.9-1,877.0) | 6,662.1  (5,158.1-8,625.3) | 4,963.4  (4,537.7-5,391.8) | 4,458.8  (4,195.1-4,734.1) | 4,588.4  (4,149.4-5,102.2) | 4,256.8  (3,864.8-4,688.7) |
| **6-11 months** | 4,766.8  (4,271.2-5,349.9) | 5,809.4  (5,267.4-6,410.1) | 6,992.0  (6,408.1-7,581.4) | 2,372.8  (1,898.2-2,841.7) | 10,109.8  (7,829.9-13,084.4) | 7,525.5  (6,880.8-8,175.5) | 6,761.7  (6,361.3-7,178.3) | 6,955.4  (6,286.8-7,737.6) | 6,453.6  (5,858.7-7,107.5) |
| **0-11 months** | 3,963.0  (3,550.4-4,448.4) | 4,830.0  (4,380.5-53,30.9) | 5,813.7  (5,328.2-6,304.8) | 1,973.2  (1,578.9-2,363.6) | 84,01.3  (6505.9-10,874.8) | 6,255.9  (5,719.8-6,796.2) | 5,620.6  (5,287.9-5,967.2) | 5,782.5  (5,227.7-6,431.7) | 5,365.0  (4,870.7-5,908.9) |
| **12-23 months** | 4,288.4  (3,840.4-4,812.8) | 5,229.7  (4,741.8-5,770.5) | 6,295.5  (5,769.8-6,825.9) | 2,133.0  (1,707.1-2,555.2) | 9,100.3  (7,041.7-11,763.2) | 6,775.4  (6,193.8-7,358.0) | 6,087.3  (5,725.9-6,462.1) | 6,259.8  (5,657.4-6,962.0) | 5,836.4  (5,294.9-6,430.5) |
| **2-4 years** | 2,012.2  (1,801.7-2,258.5) | 2,453.5  (2,224.8-2,706.5) | 2,953.6  (2,707.2-3,202.5) | 1,000.7  (800.6-1,197.3) | 4,266.1  (3,300.7-5,514.3) | 3,178.4  (2,905.5-3,452.1) | 2,855.8  (2,686.2-3,031.5) | 2,937.1  (2,654.2-3,266.6) | 2,797.7  (2,523.5-3,103.9) |
| **≥5 years** | **220.5**  **(199.3-238.4)** | **230.8**  **(210.0-250.0)** | **288.5**  **(270.2-305.4)** | **94.4**  **(75.8-110.3)** | **361.5**  **(280.3-467.8)** | **488.3**  **(443.5-531.1)** | **285.3**  **(271.9-298.4)** | **355.3**  **(325.3-383.7)** | **297.7**  **(271.3-324.1)** |
| **5-14 years** | 374.2  (338.0-404.5) | 379.5  (345.2-410.8) | 433.3  (406.3-458.0) | 258.3  (206.5-300.8) | 534.2  (413.2-691.1) | 506.9  (475.0-534.0) | 436.5  (418.2-454.6) | 490.3  (448.6-528.7) | 440.7  (403.2-478.4) |
| **15-49 years** | 140.4  (126.8-151.8) | 145.2  (132.1-157.2) | 187.7  (175.5-199.0) | 49.7  (39.9-58.0) | 204.8  (158.5-265.2) | 417.5  (372.3-462.0) | 183.2  (173.5-192.4) | 241.9  (221.4-261.8) | 198.4(  179.9-216.8) |
| **50-64 years** | 232.0  (210.1-251.0) | 240.0  (218.6-260.9) | 310.6  (290.9-328.9) | 83.7  (67.9-100.2) | 339.9  (269.8-439.3) | 688.9  (615.2-763.0) | 302.4  (286.8-318.0) | 400.7  (368.2-433.1) | 344.6  (314.0-376.0) |
| **65+ years** | 295.3  (267.3-319.2) | 305.5  (279.8-331.3) | 395.4  (369.9-419.0) | 120.1  (104.8-150.8) | 432.1  (346.1-561.2) | 879.4  (785.6-973.2) | 385.6  (365.0-405.9) | 510.7  (469.0-552.4) | 449.2  (410.4-488.9) |
| **All ages** | **540.0**  **(485.2-598.2)** | **756.7**  **(686.8-831.0)** | **839.2**  **(774.1-903.6)** | **265.6**  **(212.7-315.6)** | **1079.5**  **(835.9-1396.1)** | **1183.2**  **(1079.3-1285.7)** | **887.9**  **(838.2-938.8)** | **1,018.1**  **(923.6-1,123.0)** | **847.1**  **(768.3-932.0)** |

*Rate per 100,000 persons
