# Supplementary material for: National burden of hospitalized and non‐hospitalized influenza‐associated severe acute respiratory illness in Kenya, 2012‐2014
Source: Influenza Other Respir Viruses. 2017 Dec 15;12(1):30–7. doi: 10.1111/irv.12488 (PMC5818348; doi:10.1111/irv.12488)
Supplement: Supplementary file 4 [file IRV-12-30-s004.docx]

**S4 Table:** Annual regional rate of hospitalized influenza-associated severe acute respiratory illness (SARI) in Kenya by region, 2012 to 2014

| **Year** | **Central**  **n(95% CI)** | **Coast**  **n(95% CI)** | **Eastern**  **n(95% CI)** | **Nairobi**  **n(95% CI)** | **North Eastern**  **n(95% CI)** | **Nyanza**  **n(95% CI)** | **Rift Valley**  **n(95% CI)** | **Western**  **n(95% CI)** | **Kenya**  **n(95% CI)** |
| --- | --- | --- | --- | --- | --- | --- | --- | --- | --- |
| **2012** |  |  |  |  |  |  |  |  |  |
| **<5 years** | **141.1**  **(126.4-158.1)** | **125.2**  **(113.4-138.1)** | **184.5**  **(169.0-200.0)** | **64.8**  **(52.2-77.8)** | **98.5**  **(76.5-127.2)** | **88.0**  **(80.4-95.6)** | **184.1**  **(173.1-195.4)** | **92.9**  **(84.0-103.3)** | **135.3**  **(123.4-148.2)** |
| **<2 years** | 221.9  (199.0-248.8) | 195.1  (176.7-215.2) | 292.1  (267.5-316.4) | 98.5  (79.5-118.7) | 177.8  (138.6-229.1) | 138.3  (126.4-150.3) | 291.7  (274.2-309.5) | 145.8  (131.9-161.9) | 213.6  (195.0-233.6) |
| **0-5 months** | 191.1  (170.8-213.1) | 167.7  (151.6-185.4) | 250.3  (229.5-271.2) | 84.5  (68.8-102.2) | 154.2  (120.9-195.9) | 118.7  (108.4-129.0) | 250.7  (235.5-266.0) | 125.7  (113.7-138.9) | 183.6  (167.6-200.5) |
| **6-11 months** | 237.5  (212.6-265.8) | 209.0  (188.5-229.6) | 311.9  (285.7-338.1) | 106.2  (85.0-127.5) | 188.4  (147.4-245.7) | 147.8  (135.0-160.7) | 312.1  (293.4-331.4) | 155.2  (141.1-172.9) | 228.4  (208.5-249.8) |
| **0-11 months** | 214.5  (191.9-239.7) | 188.5  (170.2-207.7) | 281.4  (257.9-304.9) | 95.5  (77.0-115.0) | 171.5  (134.3-221.0) | 133.4  (121.8-145.0) | 281.7  (264.7-299.0) | 140.6  (127.6-156.0) | 206.2  (188.2-225.4) |
| **12-23 months** | 230.2  (206.9-259.1) | 202.7  (184.3-223.9) | 303.6  (277.8-328.7) | 102.4  (82.7-123.4) | 185.0  (143.4-238.2) | 144.0  (131.6-156.4) | 303.9  (285.8-322.4) | 151.5  (136.6-168.4) | 222.2  (202.8-243.1) |
| **2-4 years** | 89.3  (80.0-100.0) | 78.5  (71.0-86.5) | 117.4  (107.5-127.4) | 39.7  (32.0-47.4) | 70.9  (55.0-91.7) | 55.7  (50.9-60.5) | 117.6  (110.6-124.8) | 58.6  (53.0-65.3) | 86.5  (78.7-94.9) |
| **≥5 years** | **8.0**  **(7.2-8.6)** | **6.6**  **(6.0-7.1)** | **8.5**  **(7.9-9.0)** | **2.6**  **(2.2-3.1)** | **5.1**  **(4.0-6.5)** | **9.9**  **(9.0-10.8)** | **7.4**  **(7.1-7.8)** | **9.8**  **(9.0-10.5)** | **7.6**  **(7.0-8.2)** |
| **5-14 years** | 12.4  (11.2-13.4) | 10.0  (9.1-10.8) | 11.7  (11.0-12.3) | 6.7  (5.3-7.8) | 7.0  (5.4-9.1) | 9.3  (8.7-9.8) | 10.6  (10.1-11.0) | 12.4  (11.3-13.4) | 10.4  (9.6-11.2) |
| **15-49 years** | 5.4  (5.0-5.9) | 4.5  (4.1-4.8) | 5.9  (5.6-6.3) | 1.5  (1.2-1.8) | 3.2  (2.5-4.0) | 9.0  (8.0-9.9) | 5.2  (4.9-5.5) | 7.2  (6.6-7.8) | 5.5  (5.0-5.9) |
| **50-64 years** | 6.5  (5.9-7.0) | 5.2  (4.8-5.7) | 7.0  (6.6-7.5) | 2.1  (2.1-2.8) | 4.4  (3.5-5.3) | 10.8  (9.6-11.6) | 6.2  (5.8-6.5) | 8.7  (8.0-9.4) | 6.9  (6.4-7.5) |
| **65+ years** | 15.4  (13.7-16.6) | 12.8  (11.8-13.7) | 16.8  (15.4-17.8) | 5.2  (5.2-5.2) | 9.6  (7.7-11.5) | 24.9  (22.5-27.7) | 14.5  (13.9-15.5) | 19.9  (18.8-21.7) | 16.8  (15.4-18.1) |
| **All ages** | **24.3**  **(21.8-26.9)** | **25.8**  **(23.4-28.3)** | **33.6**  **(31.0-36.3)** | **10.5**  **(8.5-12.6)** | **18.3**  **(14.3-23.6)** | **23.5**  **(21.4-25.5)** | **36.2**  **(34.1-38.3)** | **24.3**  **(22.1-26.8)** | **27.3**  **(24.9-29.8)** |
|  |  |  |  |  |  |  |  |  |  |
| **2013** |  |  |  |  |  |  |  |  |  |
| **<5 years** | **96.3**  **(86.2-107.8)** | **91.2**  **(82.9-100.7)** | **137.3**  **(126.0-148.8)** | **48.2**  **(38.4-57.4)** | **77.2**  **(59.7-99.4)** | **78.7**  **(71.9-85.4)** | **153.7**  **(144.5-163.2)** | **96.2**  **(86.9-107.1)** | **110.0**  **(100.4-120.4)** |
| **<2 years** | 126.3  (113.0-141.3) | 118.7  (107.9-131.2) | 180.5  (165.8-195.8) | 62.0  (49.3-73.7) | 109.4  (85.0-140.2) | 102.9  (94.2-111.7) | 201.7  (189.6-214.0) | 125.7  (113.7-140.2) | 144.3  (131.8-157.6) |
| **0-5 months** | 52.7  (46.1-57.6) | 48.7  (44.0-53.4) | 73.4  (67.7-80.1) | 26.8  (21.1-30.6) | 44.6  (36.5-56.8) | 41.9(  39.1-45.5) | 82.8  (77.5-87.6) | 51.3  (46.6-57.1) | 59.2  (54.2-64.4) |
| **6-11 months** | 177.9  (160.1-200.5) | 168.  1(152.7-186.6) | 254.9  (233.9-276.0) | 88.4  (69.6-105.3) | 155.5  (119.6-199.4) | 145.7  (133.2-158.3) | 286.2  (269.0-303.9) | 177.5  (160.3-198.1) | 204.3  (186.7-223.4) |
| **0-11 months** | 115.9  (103.6-129.7) | 108.9  (98.8-120.6) | 165.0  (151.5-178.9) | 57.9  (45.5-68.3) | 100.6  (78.4-128.7) | 94.3  (86.6-102.4) | 185.4  (174.1-196.7) | 115.0  (104.0-128.3) | 132.4  (121.0-144.6) |
| **12-23 months** | 137.9  (123.4-154.3) | 130.1  (118.4-143.5) | 197.1  (180.9-213.8) | 67.3  (54.1-80.5) | 119.3  (92.3-153.1) | 113.0  (103.1-122.4) | 221.7  (208.5-235.2) | 137.5  (124.2-153.2) | 157.9  (144.3-172.5) |
| **2-4 years** | 77.0  (69.0-86.4) | 72.8  (66.1-80.3) | 110.3  (101.1-119.5) | 37.8  (30.4-45.3) | 66.1  (50.9-85.3) | 63.1  (57.6-68.5) | 124.0  (116.6-131.7) | 77.0  (69.6-85.6) | 88.7  (80.9-97.3) |
| **≥5 years** | **8.9**  **(8.0-9.6)** | **6.2**  **(5.7-6.8)** | **7.8**  **(7.3-8.3)** | **2.6**  **(2.1-3.0)** | **5.2**  **(4.1-6.7)** | **5.7**  **(5.2-6.2)** | **5.7**  **(5.4-5.9)** | **5.5**  **(5.1-6.0)** | **6.1**  **(5.6-6.6)** |
| **5-14 years** | 18.9  (17.1-20.4) | 12.5  (11.4-13.5) | 14.6  (13.7-15.5) | 8.4  (6.8-9.7) | 8.7  (6.7-11.3) | 7.8  (7.4-8.2) | 10.5  (10.1-10.9) | 9.4  (8.6-10.1) | 11.3  (10.4-12.2) |
| **15-49 years** | 4.5  (4.0-4.8) | 3.0  (2.7-3.3) | 4.0  (3.7-4.2) | 1.0  (0.8-1.2) | 2.1  (1.7-2.8) | 4.0  (3.6-4.5) | 2.8  (2.6-2.9) | 2.9  (2.7-3.1) | 3.1  (2.8-3.4) |
| **50-64 years** | 9.6  (8.5-10.4) | 6.5  (6.0-7.0) | 8.4  (8.0-9.1) | 2.7  (2.1-2.7) | 5.2  (4.3-6.0) | 8.6  (7.7-9.7) | 5.8  (5.5-6.2) | 6.4  (5.7-6.7) | 7.1  (6.5-7.7) |
| **65+ years** | 5.3  (4.9-5.7) | 3.5  (3.5-4.4) | 4.6  (4.2-4.9) | 2.6  (2.6-2.6) | 3.7  (3.7-3.7) | 4.6  (4.1-5.5) | 3.4  (3.1-3.4) | 3.3  (3.3-3.9) | 4.1  (3.8-4.5) |
| **All ages** | **19.5**  **(17.6-21.6)** | **20.0**  **(18.2-22.0)** | **26.3**  **(24.3-28.4)** | **8.4**  **(6.7-9.9)** | **15.4**  **(12.0-19.9)** | **18.4**  **(16.8-20.0)** | **29.7**  **(28.0-31.5)** | **21.4**  **(19.4-23.6)** | **22.1**  **(20.2-24.1)** |
|  |  |  |  |  |  |  |  |  |  |
| **2014** |  |  |  |  |  |  |  |  |  |
| **<5 years** | **57.4**  **(51.5-64.7)** | **78.5**  **(71.4-86.8)** | **79.2**  **(72.5-85.9)** | **25.0**  **(20.1-29.6)** | **52.8**  **(40.7-67.8)** | **53.2**  **(48.8-57.9)** | **67.4**  **(63.5-71.6)** | **36.4**  **(33.0-40.5)** | **59.3**  **(54.0-65.1)** |
| **<2 years** | 80.7  (72.7-91.2) | 109.7  (99.9-121.5) | 111.9  (102.6-121.5) | 34.8  (28.0-41.0) | 82.1  (63.4-105.0) | 74.9  (68.7-81.5) | 95.4  (89.9-101.3) | 51.3  (46.3-56.9) | 83.5  (76.3-91.7) |
| **0-5 months** | 85.4  (77.3-96.7) | 116.7  (106.0-129.0) | 119.2  (109.3-129.2) | 37.5  (30.0-43.1) | 87.4  (67.5-111.2) | 79.3  (73.0-86.4) | 101.3  (95.0-107.5) | 54.8  (49.1-60.5) | 88.7  (80.9-97.1) |
| **6-11 months** | 77.5  (69.6-88.6) | 105.6  (96.6-117.7) | 107.4  (98.7-117.1) | 33.1  (27.6-40.5) | 78.0  (62.4-101.4) | 72.6  (66.5-78.7) | 91.8  (86.7-97.4) | 49.3  (44.8-54.9) | 80.3  (73.6-88.4) |
| **0-11 months** | 81.4  (73.5-92.6) | 111.1  (101.2-123.3) | 113.3  (104.0-123.1) | 35.3  (28.8-41.8) | 82.7  (64.9-106.3) | 75.9  (69.7-82.5) | 96.5  (90.8-102.4) | 52.0  (46.9-57.7) | 84.5  (77.2-92.7) |
| **12-23 months** | 79.9  (71.9-89.7) | 108.0  (98.3-119.4) | 110.4  (101.1-119.8) | 34.1  (27.0-40.0) | 81.5  (61.7-103.5) | 73.7  (67.6-80.4) | 94.0  (88.7-100.0) | 50.6  (45.7-56.1) | 82.5  (75.2-90.5) |
| **2-4 years** | 42.5  (38.0-47.6) | 57.6  (52.4-63.6) | 58.7  (53.8-63.7) | 17.7  14.3-21.2) | 42.6  (32.9-54.9) | 39.3  (36.0-42.7) | 50.2  (47.2-53.2) | 26.8  (24.3-29.8) | 44.1  (40.1-48.6) |
| **≥5 years** | **5.1**  **(4.6-5.5)** | **4.9**  **(4.4-5.2)** | **6.2**  **(5.8-6.6)** | **2.0**  **(1.6-2.3)** | **3.7**  **(2.8-4.7)** | **4.9**  **(4.4-5.3)** | **6.1**  **(5.8-6.4)** | **5.5**  **(5.0-6.0)** | **5.2**  **(4.8-5.6)** |
| **5-14 years** | 7.3  (6.7-8.0) | 6.9  (6.3-7.4) | 8.0  (7.5-8.5) | 4.6  (3.8-5.4) | 4.8  (3.8-6.2) | 4.2  (4.0-4.4) | 8.1  (7.8-8.4) | 6.5  (5.9-7.0) | 6.7  (6.2-7.2) |
| **15-49 years** | 3.7  (3.4-4.0) | 3.5  (3.2-3.8) | 4.7  (4.4-4.9) | 1.2  (1.0-1.4) | 2.5  (1.9-3.2) | 4.7  (4.1-5.1) | 4.6  (4.3-4.8) | 4.3  (4.0-4.7) | 3.9  (3.6-4.2) |
| **50-64 years** | 6.4  (5.9-7.0) | 6.4  (5.9-6.8) | 8.2  (7.6-8.7) | 2.7  (2.0-2.7) | 5.1  (3.4-5.9) | 8.1  (7.3-8.9) | 8.0  (7.6-8.3) | 7.6  (6.9-8.3) | 7.2  (6.6-7.7) |
| **65+ years** | 6.3(  5.9-7.1) | 6.1  (6.1-6.9) | 8.0  (7.7-8.6) | 2.5  (2.5-5.0) | 5.5  (3.7-7.3) | 8.0  (7.2-8.9) | 7.8  (7.5-8.4) | 7.6  (7.1-8.1) | 7.3  (6.8-8.1) |
| **All ages** | **11.5**  **(10.4-12.8)** | **16.8**  **(15.3-18.4)** | **16.7**  **(15.4-17.9)** | **4.9**  **(4.0-5.8)** | **10.7**  **(8.2-13.7)** | **13.3**  **(12.2-14.5)** | **16.1**  **(15.2-17.0)** | **10.9**  **(9.9-12.0)** | **13.5**  **(12.3-14.8)** |
|  |  |  |  |  |  |  |  |  |  |
| **2012-2014** |  |  |  |  |  |  |  |  |  |
| **<5 years** | **97.9**  **(87.7-109.8)** | **106.4**  **(96.4-117.3)** | **133.7**  **(122.6-144.9)** | **45.1**  **(36.2-53.9)** | **78.7**  **(61.2-101.8)** | **75.9**  **(69.6-82.5)** | **130.0**  **(122.3-138.0)** | **69.5**  **(62.8-77.1)** | **100.6**  **(91.7-110.3)** |
| **<2 years** | 142.4  (127.4-159.6) | 153.5  (138.9-169.0) | 195.2  (179.0-211.7) | 63.7  (51.5-76.4) | 127.7  (100.0-164.9) | 110.4  (101.2-119.8) | 189.9  (178.6-201.6) | 100.8  (91.2-111.7) | 146.6  (133.8-160.3) |
| **0-5 months** | 108.8  (97.3-122.0) | 117.9  (106.9-128.9) | 149.2  (136.7-161.6) | 49.8  (40.3-59.4) | 97.6  (77.2-126.0) | 84.8  (77.5-92.0) | 145.1  (136.6-154.1) | 77.1  (70.1-85.2) | 112.2  (102.5-122.6) |
| **6-11 months** | 165.2  (147.4-184.6) | 177.6  (160.6-196.1) | 225.3  (207.6-245.3) | 73.4  (60.3-88.5) | 147.7  (115.8-191.7) | 128.0  (117.3-138.8) | 220.3  (207.3-233.9) | 117.0  (105.5-129.6) | 169.7  (155.1-185.8) |
| **0-11 months** | 137.3  (122.6-153.6) | 148.0  (134.0-162.8) | 187.6  (172.5-203.8) | 61.8  (50.4-74.1) | 122.9  (96.7-159.1) | 106.6  (97.6-115.6) | 183.0  (172.2-194.4) | 97.2  (87.9-107.6) | 141.2  (129.1-154.5) |
| **12-23 months** | 148.1  (132.7-166.3) | 159.9  (144.6-176.1) | 203.3  (186.0-220.1) | 66.2  (52.9-79.4) | 133.0  (103.7-171.4) | 114.7  (105.3-124.7) | 198.2  (186.3-210.4) | 104.8  (94.7-116.2) | 152.7  (139.3-167.1) |
| **2-4 years** | 69.4  (62.3-78.0) | 74.8  (67.9-82.7) | 95.3  (87.4-103.2) | 31.2  (24.9-37.1) | 61.7  (47.7-79.8) | 53.8  49.3-58.5) | 93.0  (87.4-98.7) | 49.1  (44.4-54.6) | 72.0  (65.5-79.1) |
| **≥5 years** | **7.3**  **(6.6-7.9)** | **5.8**  **(5.3-6.3)** | **7.5**  **(7.0-7.9)** | **2.4**  **(1.9-2.8)** | **4.5**  **(3.6-5.9)** | **6.5**  **(5.9-7.1)** | **6.6**  **(6.3-6.9)** | **7.1**  **(6.5-7.7)** | **6.3**  **(5.8-6.8)** |
| **5-14 years** | 12.3  (11.2-13.4) | 9.5  (8.7-10.3) | 11.2  (10.5-11.8) | 6.5  (5.2-7.5) | 6.6  (5.2-8.6) | 6.7  (6.3-7.1) | 10.1  (9.6-10.5) | 9.7  (8.9-10.5) | 9.4  (8.7-10.1) |
| **15-49 years** | 4.6  (4.2-5.0) | 3.7(3.3-3.9) | 4.8  (4.5-5.1) | 1.2  (1.0-1.5) | 2.6  (2.1-3.4) | 5.5  (4.9-6.1) | 4.2  (4.0-4.4) | 4.8  (4.4-5.2) | 4.1(  3.8-4.5) |
| **50-64 years** | 7.7(  7.1-8.5) | 6.0  (5.6-6.5) | 8.2  (7.5-8.6) | 2.1  (2.1-2.7) | 4.3  (3.5-6.1) | 9.1  (8.3-10.2) | 7.1  (6.7-7.4) | 8.1  (7.4-8.8) | 7.3  (6.7-8.0) |
| **65+ years** | 10.1  (8.9-10.5) | 8.0  (7.1-8.9) | 10.5  (9.8-10.8) | 5.1  (2.6-5.1) | 5.6  (5.6-7.5) | 11.9  (10.5-13.3) | 8.9  (8.6-9.5) | 10.6  (9.4-11.1) | 9.8  (8.9-10.5) |
| **All ages** | **18.4**  **(16.5-20.4)** | **22.1**  **(20.0-24.3)** | **25.5**  **(23.5-27.5)** | **7.8**  **(6.3-9.3)** | **15.1**  **(11.8-19.5)** | **18.6**  **(17.0-20.2)** | **26.6**  **(25.1-28.2)** | **18.0**  **(16.3-19.8)** | **20.8**  **(19.0-22.7)** |

*Rate per 100,000 persons
