# Supplementary material for: National burden of hospitalized and non‐hospitalized influenza‐associated severe acute respiratory illness in Kenya, 2012‐2014
Source: Influenza Other Respir Viruses. 2017 Dec 15;12(1):30–7. doi: 10.1111/irv.12488 (PMC5818348; doi:10.1111/irv.12488)
Supplement: Supplementary file 5 [file IRV-12-30-s005.docx]

**S5 Table:** Annual regional rate of non-hospitalized influenza-associated severe acute respiratory illness (SARI) in Kenya by region, 2012 to 2014

| **Year** | **Central**  **n(95% CI)** | **Coast**  **n(95% CI)** | **Eastern**  **n(95% CI)** | **Nairobi**  **n(95% CI)** | **North Eastern**  **n(95% CI)** | **Nyanza**  **n(95% CI)** | **Rift Valley**  **n(95% CI)** | **Western**  **n(95% CI)** | **Kenya**  **n(95% CI)** |
| --- | --- | --- | --- | --- | --- | --- | --- | --- | --- |
| **2012** |  |  |  |  |  |  |  |  |  |
| **<5 years** | **407.2**  **(365.1-456.4)** | **393.4**  **(356.2-433.5)** | **561.1**  **(513.9-608.3)** | **207.1**  **(166.9-248.4)** | **661.7**  **(514.2-854.1)** | **250.7**  **(229.2-272.3)** | **554.3**  **(521.2-588.3)** | **356.4**  **(322.3-396.1)** | **436.4**  **(395.1-482.1)** |
| **<2 years** | 640.7  (574.9-718.3) | 613.2  (555.0-675.6) | 888.2  (813.2-962.4) | 315.2  (254.2-378.9) | 1,195.8  (931.8-1,539.5) | 394.1  (360.2-428.1) | 878.2  (825.5-931.9) | 559.2  (505.9-621.1) | 682.2  (619.3-751.1) |
| **0-5 months** | 551.3  (493.8-615.6) | 527.2  (475.6-582.0) | 761.4  (697.7-825.2) | 271.3  (220.2-326.4) | 10,38.0  (812.9-1,317.3) | 338.4  (309.4-367.4) | 754.9  (709.1-800.7) | 482.5  (437.0-532.8) | 586.2  (532.3-644.0) |
| **6-11 months** | 686.0  (614.6-767.4) | 657.2  (592.3-720.6) | 948.3  (868.6-1027.9) | 339.9  (272.3-407.5) | 1265.3  (991.0-1,650.2) | 421.5  (384.7-458.2) | 939.6  (883.4-998.0) | 595.1  (541.0-663.3) | 728.3  (661.4-802.4) |
| **0-11 months** | 619.3  (554.7-692.2) | 592.8  (534.5-651.9) | 855.7  (783.9-927.5) | 305.9  (246.5-367.3) | 1,152.7  (902.7-1,485.2) | 380.3  (347.4-413.2) | 848.1  (797.1-900.2) | 539.3  (489.5-598.7) | 657.9  (597.4-723.9) |
| **12-23 months** | 664.5  (597.4-747.4) | 636.7  (578.6-703.0) | 923.0  (844.5-999.7) | 326.9  (264.0-393.6) | 1,244.1  (964.3-1,600.2) | 410.0  (375.1-445.5) | 915.1  (860.3-970.5) | 580.9  (523.9-645.7) | 710.3  (644.5-782.5) |
| **2-4 years** | 257.7  (230.8-288.7) | 246.3  (223.1-271.5) | 356.9  (327.1-387.3) | 126.7  (102.0-151.4) | 476.1  (369.2-616.0) | 158.5  (144.9-172.1) | 354.0  (332.9-375.8) | 224.9  (203.2-250.2) | 283.2  (255.5-314.5) |
| **≥5 years** | **42.5**  **(38.5-45.9)** | **37.6**  **(34.4-40.6)** | **47.2**  **(44.2-49.9)** | **15.3**  **(12.6-18.1)** | **58.6**  **(45.8-74.8)** | **52.0**  **(47.2-56.6)** | **41.1**  **(39.2-43.0)** | **66.9**  **(61.4-72.3)** | **45.1**  **(41.1-49.1)** |
| **5-14 years** | 65.9  (59.8-71.1) | 57.2  (52.3-61.9) | 65.0  (61.0-68.6) | 38.8  (31.0-45.5) | 81.1  (62.7-104.5) | 48.8  (45.6-51.3) | 58.3  (55.9-60.6) | 85.1  (77.7-91.5) | 62.6  (57.1-68.1) |
| **15-49 years** | 28.9  (26.3-31.3) | 25.8  (23.5-27.7) | 33.0  (31.0-34.9) | 8.8  (7.2-10.4) | 36.5  (28.7-46.5) | 47.1  (41.9-52.1) | 28.7  (27.1-30.2) | 49.3  (45.2-53.4) | 31.8  (28.9-34.7) |
| **50-64 years** | 34.6  (31.5-37.5) | 30.1  (27.7-32.9) | 39.3  (36.8-41.8) | 12.7  (12.7-16.9) | 51.4  (41.7-62.1) | 56.7  (50.7-61.2) | 34.2  (32.2-36.2) | 59.6  (54.7-64.4) | 40.6  (37.2-44.1) |
| **65+ years** | 81.9  (73.2-88.6) | 73.8  (68.3-78.3) | 93.7  (85.9-99.0) | 31.4  (31.4-31.4) | 111.3  (90.2-134.3) | 130.9  (118.7-145.5) | 80.2  (76.7-85.5) | 136.8  (129.4-148.7) | 97.0  (89.3-105.1) |
| **All ages** | **87.1**  **(78.5-96.1)** | **95.2**  **(86.5-104.2)** | **120.6**  **(111.4-129.7)** | **39.6**  **(32.1-47.2)** | **144.3**  **(112.3-185.5)** | **86.6**  **(78.9-94.2)** | **124.5**  **(117.5-131.7)** | **117.5**  **(107.1-128.9)** | **105.3**  **(95.6-115.7)** |
|  |  |  |  |  |  |  |  |  |  |
| **2013** |  |  |  |  |  |  |  |  |  |
| **<5 years** | **278.1**  **(248.9-311.3)** | **286.3**  **(260.3-316.4)** | **417.6**  **(383.1-452.6)** | **154.0**  **(123.0-183.6)** | **518.8**  **(401.6-667.8)** | **224.0**  **(205.0-243.3)** | **462.7**  **(435.1-491.2)** | **369.0**  **(333.5-410.7)** | **356.3**  **(323.0-393.2)** |
| **<2 years** | 365.2  (326.5-408.2) | 372.9  (339.1-412.5) | 549.3  (504.2-595.5) | 198.2  (158.0-235.9) | 735.1  (572.6-942.3) | 293.1  (268.6-318.3) | 607.4  (571.0-644.4) | 482.4  (436.3-537.9) | 461.9  (420.1-507.8) |
| **0-5 months** | 153.1  (133.4-166.3) | 153.9  (138.2-168.0) | 223.4  (206.5-243.7) | 86.2  (68.9-99.6) | 300.4  (247.6-381.5) | 119.2  (112.0-130.2) | 249.5  (233.5-263.8) | 197.0  (179.5-219.2) | 189.6  (173.0-207.4) |
| **6-11 months** | 514.3  (462.6-579.0) | 527.4  (479.6-586.0) | 775.9  (711.6-839.1) | 282.1  (221.9-336.6) | 1,044.6  (805.4-1,339.7) | 414.9  (380.0-450.6) | 861.7  (810.1-914.9) | 681.4  (615.0-760.4) | 653.5  (593.9-718.9) |
| **0-11 months** | 335.4  (299.5-374.5) | 342.3  (310.4-378.9) | 502.1  (461.3-544.1) | 185.0  (146.1-219.1) | 675.8  (529.0-864.9) | 268.  4(247.2-291.8) | 558.3  (524.4-592.3) | 441.4  (399.2-492.2) | 423.6  (385.3-465.5) |
| **12-23 months** | 398.4  (356.6-445.6) | 408.2  (372.3-451.3) | 599.6  (550.0-650.3) | 215.0  (173.0-257.1) | 801.6  (621.4-1029.0) | 321.8  (293.5-349.0) | 667.4  (627.8-708.2) | 527.2  (476.7-587.7) | 506.2  (460.2-556.8) |
| **2-4 years** | 222.3  (199.2-249.2) | 228.4  (207.5-252.1) | 335.4  (307.5-363.4) | 121.0  (97.0-144.7) | 443.6  (342.1-572.5) | 179.6  (164.2-195.1) | 373.2  (351.1-396.4) | 295.4  (266.9-328.3) | 290.5  (262.5-321.8) |
| **≥5 years** | **47.1**  **(42.5-50.9)** | **35.7**  **(32.5-38.8)** | **43.5**  **(40.8-46.1)** | **15.3**  **(12.4-17.6)** | **60.3**  **(47.2-77.5)** | **30.0**  **(27.5-32.6)** | **31.2**  **(29.7-32.6)** | **37.8**  **(34.7-40.9)** | **36.3**  **(33.0-39.7)** |
| **5-14 years** | 100.3(  90.7-108.5) | 71.6  (65.1-77.5) | 81.6  (76.4-86.1) | 49.0  (39.6-56.6) | 100.5  (77.8-130.4) | 41.1  (38.6-43.2) | 57.8  (55.5-60.2) | 64.2  (59.0-69.5) | 67.9  (61.7-74.3) |
| **15-49 years** | 23.7  (21.4-25.6) | 17.2  (15.6-18.7) | 22.2  (20.8-23.5) | 6.0(  4.9-7.0) | 24.8  (19.4-32.4) | 21.3  (18.9-23.6) | 15.3  (14.4-16.0) | 19.9  (18.2-21.5) | 18.2  (16.5-19.9) |
| **50-64 years** | 51.0  (45.2-55.4) | 37.6  (34.8-39.9) | 46.9  (44.4-50.6) | 16.4  (12.3-16.4) | 60.5  (50.1-69.9) | 45.0  (40.8-50.8) | 32.4  (30.5-34.4) | 44.2  (39.5-46.6) | 41.7  (37.9-45.2) |
| **65+ years** | 28.4  (25.9-30.4) | 20.4  (20.4-25.7) | 25.5  (23.9-27.5) | 15.3  (15.3-15.3) | 44.8  (44.8-44.8) | 24.2(21.9-29.2) | 18.7  (17.2-18.7) | 23.3  (23.3-26.6) | 24.1  (22.7-26.4) |
| **All ages** | **75.3**  **(67.7-82.7)** | **76.3**  **(69.4-83.7)** | **96.**  **9(89.7-104.2)** | **32.9**  **(26.4-38.6)** | **125.4**  **(97.5-161.4)** | **63.8**  **(58.4-69.3)** | **101.3**  **(95.6-107.1)** | **95.7**  **(86.9-105.5)** | **85.5**  **(77.6-94.1)** |
|  |  |  |  |  |  |  |  |  |  |
| **2014** |  |  |  |  |  |  |  |  |  |
| **<5 years** | **165.9**  **(148.8-186.9)** | **246.5**  **(224.4-272.6)** | **240.8**  **(220.6-261.4)** | **80.1**  **(64.6-95.1)** | **355.1**  **(273.9-455.6)** | **151.7**  **(139.0-164.9)** | **203.0**  **(191.2-215.7)** | **139.8**  **(126.5-155.4)** | **192.7**  **(173.9-213.9)** |
| **<2 years** | 233.3  (210.2-264.0) | 344.5  (313.9-381.6) | 340.5  (311.9-369.9) | 111.5  (90.2-131.7) | 553.0  (427.2-705.8) | 213.5  (196.0-232.5) | 287.1  (270.6-305.2) | 197.1  (177.9-218.6) | 268.0  (242.9-296.3) |
| **0-5 months** | 246.5  (224.0-280.3) | 367.1  (333.3-405.5) | 363.3  (332.4-393.1) | 119.9  (97.4-138.6) | 587.8  (456.7-746.7) | 226.2  (208.4-246.7) | 304.8  (286.1-324.1) | 209.9  (188.2-232.7) | 284.3  (257.6-313.7) |
| **6-11 months** | 224.7  (201.0-256.4) | 331.9  (303.3-369.7) | 326.5  (300.4-356.8) | 106.7  (88.3-130.6) | 526.7  (421.3-682.7) | 207.3  (189.8-224.8) | 276.5  (261.2-293.3) | 189.4  (172.6-210.7) | 257.3  (234.4-285.5) |
| **0-11 months** | 235.5  (212.4-268.3) | 349.4  (318.2-387.4) | 344.7  (316.2-374.8) | 113.2  (92.8-134.6) | 557.0  (438.9-714.4) | 216.7  (199.0-235.7) | 290.5  (273.5-308.5) | 199.5  (180.3-221.6) | 270.6  (245.9-299.5) |
| **12-23 months** | 230.9  (207.8-259.3) | 338.8  (309.0-374.8) | 336.0  (307.4-364.6) | 109.3  (87.0-128.1) | 548.6  (414.2-696.2) | 209.9  (192.5-228.8) | 283.0  (266.9-301.2) | 194.4  (175.2-215.3) | 264.9  (239.5-292.6) |
| **2-4 years** | 122.7  (109.6-137.5) | 180.9  (164.5-199.7) | 178.7  (163.7-193.6) | 56.7  (45.5-67.9) | 286.4  (220.7-368.7) | 112.0  (102.4-121.5) | 151.0  (142.1-160.3) | 102.7  (93.2-114.4) | 145.8  (130.9-162.5) |
| **≥5 years** | **27.0**  **(24.6-29.4)** | **27.9**  **(25.5-30.0)** | **34.7**  **(32.5-36.7)** | **11.6**  **(9.4-13.5)** | **42.6**  **(32.9-54.8)** | **25.7**  **(23.4-28.0)** | **33.7**  **(32.2-35.3)** | **37.6**  **(34.6-40.8)** | **30.6**  **(27.9-33.3)** |
| **5-14 years** | 39.1  (35.4-42.6) | 39.4  (35.9-42.2) | 44.6  (41.8-47.1) | 26.8  (22.2-31.4) | 55.1  (43.3-71.4) | 22.0  (20.9-23.2) | 44.8  (42.9-46.6) | 44.6  (40.8-48.3) | 40.3  (36.9-44.0) |
| **15-49 years** | 19.7  (17.8-21.4) | 20.2  (18.3-21.7) | 26.0  (24.3-27.5) | 7.1  (5.6-8.1) | 28.5  (22.2-36.9) | 24.5  (21.8-26.9) | 25.1  (23.9-26.5) | 29.5  (27.2-32.1) | 22.7  (20.7-24.7) |
| **50-64 years** | 34.3  (31.4-37.3) | 36.8  (34.1-39.1) | 45.9  (42.2-48.2) | 16.1  (12.1-16.1) | 59.1  (39.7-68.4) | 42.6  (38.3-47.0) | 44.0  (42.1-45.9) | 52.2  (47.5-56.8) | 42.2  (38.1-45.2) |
| **65+ years** | 33.7  (31.7-38.0) | 35.6  (35.6-39.9) | 44.8  (42.9-48.3) | 15.0  (15.0-30.0) | 64.0  (43.9-85.9) | 42.5  (38.0-47.4) | 43.3  (41.5-46.6) | 52.1  (48.9-55.9) | 42.4  (39.5-47.2) |
| **All ages** | **44.0**  **(39.7-48.7)** | **63.3**  **(57.7-69.3)** | **64.2**  **(59.4-68.8)** | **20.3**  **(16.4-23.8)** | **87.0**  **(67.1-111.8)** | **47.7**  **(43.5-51.9)** | **61.2(58.0-64.6)** | **55.5**  **(50.7-60.9)** | **55.5**  **(50.4-61.1)** |
|  |  |  |  |  |  |  |  |  |  |
| **2012-2014** |  |  |  |  |  |  |  |  |  |
| **<5 years** | **282.8**  **(253.3-317.1)** | **334.1**  **(302.8-368.2)** | **406.6**  **(372.9-440.7)** | **144.0**  **(115.9-172.1)** | **529.4**  **(411.2-684.1)** | **216.3**  **(198.2-235.0)** | **391.5**  **(368.1-415.5)** | **266.5**  **(241.0-295.8)** | **325.7**  **(294.7-360.3)** |
| **<2 years** | 411.8  (368.3-460.8) | 482.3  (436.8-530.7) | 594.0  (544.5-643.7) | 203.8  (165.1-244.2) | 859.6  (672.4-1109.6) | 314.6  (288.2-341.5) | 571.8  (537.6-607.0) | 386.8  (349.6-428.7) | 469.0  (425.7-516.6) |
| **0-5 months** | 315.0  (282.0-352.9) | 371.0  (336.4-405.6) | 454.3  (415.9-491.6) | 159.1  (128.5-189.8) | 658.5  (520.3-849.5) | 241.6  (220.6-262.5) | 436.9  (411.4-464.0) | 295.4  (268.5-326.9) | 358.7  (326.0-394.7) |
| **6-11 months** | 477.8  (426.0-532.9) | 557.5  (505.0-616.1) | 686.0  (631.6-745.9) | 235.4  (194.0-282.5) | 994.2  (778.6-1289.7) | 365.3  (334.0-395.7) | 663.5  (623.9-704.3) | 448.4  (404.8-497.7) | 542.6  (493.0-598.1) |
| **0-11 months** | 397.1  (354.6-443.7) | 465.1  (421.5-511.8) | 571.2  (524.7-619.9) | 197.6  (161.5-236.6) | 827.8  (650.6-1071.6) | 304.0(277.8-329.7) | 551.2  (518.6-585.2) | 372.6  (337.3-413.1) | 451.5  (410.3-497.3) |
| **12-23 months** | 428.0  (383.5-479.8) | 502.2  (454.6-552.5) | 618.4  (565.7-669.2) | 211.7  (169.6-253.8) | 895.2  (696.7-1152.2) | 327.0  (300.2-355.2) | 596.9  (560.8-633.6) | 402.2  (363.1-445.8) | 489.2(443.4-538.8) |
| **2-4 years** | 200.3  (179.6-225.1) | 234.8  (213.1-259.4) | 289.7  (265.7-313.9) | 99.5  (79.3-118.4) | 414.7  (320.5-536.3) | 153.2  (140.4-166.6) | 279.9  (263.2-297.1) | 188.6  (170.5-209.5) | 236.5  (213.0-262.9) |
| **≥5 years** | **38.8**  **(35.2-42.1)** | **33.3**  **(30.4-36.1)** | **41.7**  **(39.0-44.0)** | **14.0**  **(11.1-16.3)** | **52.4**  **(41.5-68.2)** | **34.0**  **(30.9-37.1)** | **36.3**  **(34.7-38.0)** | **48.7**  **(44.4-52.5)** | **37.3**  **(34.0-40.7)** |
| **5-14 years** | 65.6  (59.6-71.1) | 54.5  (49.8-59.3) | 62.4  (58.5-65.9) | 37.8  (30.2-43.5) | 76.7  (59.9-99.5) | 35.4  (32.9-37.2) | 55.5  (53.1-57.8) | 66.7  (61.0-72.0) | 56.7  (51.6-61.8) |
| **15-49 years** | 24.6  (22.3-26.7) | 21.0  (19.1-22.6) | 27.0  (25.3-28.5) | 7.3  (5.7-8.6) | 30.2  (23.7-38.9) | 28.9  (25.8-32.1) | 23.3  (22.1-24.4) | 33.2  (30.2-35.8) | 24.1(21.9-26.3) |
| **50-64 years** | 40.9  (38.2-45.3) | 34.9  (32.1-37.7) | 45.6  (42.1-48.3) | 12.4  (12.4-16.5) | 50.2  (40.6-70.0) | 48.1  (43.6-53.9) | 39.2  (37.2-41.2) | 55.7  (51.0-60.5) | 42.4  (39.1-46.7) |
| **65+ years** | 53.9  (47.5-56.4) | 46.2  (40.9-51.5) | 58.6  (54.7-60.2) | 30.7  (15.3-30.7) | 65.5  (65.5-87.9) | 62.7  (55.4-70.0) | 49.2  (47.7-52.9) | 72.8  (65.0-76.7) | 56.7  (51.6-61.0) |
| **All ages** | **68.6**  **(61.9-75.7)** | **82.0**  **(74.4-89.9)** | **93.8**  **(86.7-100.7)** | **30.5**  **(24.4-36.0)** | **120.2**  **(94.0-155.7)** | **65.8**  **(60.0-71.6)** | **94.1**  **(88.9-99.4)** | **86.8**  **(78.8-95.1)** | **81.7**  **(74.1-89.9)** |

*Rate per 100,000 persons
