# Supplementary material for: National burden of hospitalized and non‐hospitalized influenza‐associated severe acute respiratory illness in Kenya, 2012‐2014
Source: Influenza Other Respir Viruses. 2017 Dec 15;12(1):30–7. doi: 10.1111/irv.12488 (PMC5818348; doi:10.1111/irv.12488)
Supplement: Supplementary file 6 [file IRV-12-30-s006.docx]

**S6 Table:** Differences in methodology between original study and current study

| **Difference in method** | **Original study** | **Current study** |
| --- | --- | --- |
| Catchment population of SCRH (hospital within base region) for calculation of base SARI rates | Population enrolled in HDSS within 5 km of SCRH in Karemo division | Population enrolled in HDSS residing in Karemo division |
| Estimation of HIV prevalence in children | Algorithm developed by authors that took into account regional differences in prevalence of HIV positive mothers, enrollment in prevention to mother to child transmission programs, and HIV mother to child transmission rates | Number of HIV positive children 0-14 years of age in each region divided by number of children of 0-14 years of age in each region |
| Calculation of rates of non-hospitalized influenza-associated SARI | Used health care utilization findings on proportion of individuals with pneumonia who visited a hospital in the past 12 months | Used health care utilization findings on proportion of individuals with pneumonia who were admitted in the past 12 months |
