# Supplementary material for: National burden of hospitalized and non‐hospitalized influenza‐associated severe acute respiratory illness in Kenya, 2012‐2014
Source: Influenza Other Respir Viruses. 2017 Dec 15;12(1):30–7. doi: 10.1111/irv.12488 (PMC5818348; doi:10.1111/irv.12488)
Supplement: Supplementary file 7 [file IRV-12-30-s007.docx]

**S7 Table:** Risk factors, relative risks values and sources of data for the prevalence of risk factors in each region

| **Risk factor** | **Relative risk (reference number)** | **Source of risk factor prevalence data in the original study (reference number)** | **Source of risk factor prevalence data in the current study (reference number)** |
| --- | --- | --- | --- |
| Malnutrition (weight for age Z-score ≤2) | 1.8 (4) | Kenya Demographic Health Survey (DHS) 2009 (5) | Kenya DHS 2014 report and dataset (6, 7) |
| Low birth weight (<2500 g) | 1.4 (4) | Kenya DHS 2009 (5) | Kenya DHS 2014 report and dataset (6, 7) |
| Non-exclusive breastfeeding (during first 4 months of life) | 1.9 (4) | MICS UNICEF 2000 (8) | Kenya DHS 2014 dataset (7) |
| Household air pollution (use of solid fuels for cooking) | 1.8 (4) | Kenya DHS 2009 (5) | Kenya DHS 2014 report (6) and Kenya population census 2009 (9) |
| Crowding (≥ 5 per household) | 1.4 (4) | Kenya DHS 2003 (10) | Kenya DHS 2014 dataset (7) |
| HIV prevalence  (children 0 - 14 years) | 7.2 (11) | Algorithm provided in Appendix 2 of original paper (1) | Kenya HIV estimates 2014 (12) |
| HIV prevalence  (≥ 15 years) | 5.64 (13) | Kenya DHS 2009 (5) | KAIS 2012 (14) and Kenya population census 2009 (9) |

NB: References follow the numbering in the supplementary text
