# Supplementary material for: National burden of hospitalized and non‐hospitalized influenza‐associated severe acute respiratory illness in Kenya, 2012‐2014
Source: Influenza Other Respir Viruses. 2017 Dec 15;12(1):30–7. doi: 10.1111/irv.12488 (PMC5818348; doi:10.1111/irv.12488)
Supplement: Supplementary file 8 [file IRV-12-30-s008.docx]

# Supplementary information on methodology

## Equations used in calculating the burden of influenza-associated severe acute respiratory illness

The method and equations presented in the following section are cited from the methodology described by Fuller *et al* (1).

1. Annual base rates of hospitalized severe acute respiratory illness (SARI) for the years 2012 to 2014 were calculated for the population of Karemo division enrolled in the Health and Demographic Surveillance System (HDSS). The number of hospitalized SARI patients at Siaya County Referral Hospital (SCRH) was divided by the age-specific population of HDSS residents in Karemo Division. The base rate of hospitalized SARI was calculated for each of the age groups and years under study.

$$I_{B}=\frac{{SARI}_{B}}{{Pop}_{B}}$$

Equation 1

Where:

$I_{B}$ = Base rate of hospitalized SARI

${SARI}_{B}$ = Total number of cases meeting SARI case definition hospitalized in base region per year

${Pop}_{B}$ = Population of surveillance catchment area

1. An adjustment factor for hospitalized SARI within each region was calculated. The net prevalence of risk factors within each region (after deduction of the base region prevalence), was multiplied by each risk factor’s relative risk value and summed. This figure was multiplied by the health care seeking practices in each region divided by the base region to account for differences in health care seeking practices between regions.

$${Adj}_{Y}=\left( 1+\sum_{i} \left( P_{i,Y}-P_{i,B} \right)\times\left( {RR}_{i}-1 \right) \right)\times\frac{{DHS}_{Y}}{{DHS}_{B}}$$

Equation 2

Where:

${Adj}_{Y}$ = Adjustment factor for region Y

$P_{i,Y}$ = Prevalence of risk factor *i* in region Y

$P_{i,B}$ = Prevalence of risk factor *i* in base region

${RR}_{i}$ = Relative risk of SARI due to risk factor *i*

${DHS}_{Y}$ = Proportion of ARI cases seeking care in region Y (from DHS)

${DHS}_{B}$ = Proportion of ARI cases seeking care in base region (from DHS)

1. The incidence of hospitalized SARI in each region was calculated by multiplying the base rate of hospitalized SARI by the adjustment factor for the region

$$I_{H,Y}=I_{B} \times{Adj}_{Y}$$

Equation 3

Where:

$I_{H,Y}$ = Incidence of hospitalized SARI in region Y

1. The rates of non-hospitalized SARI in the base region were calculated by applying findings from a health care utilization survey specific to hospitalization for pneumonia (2). The proportion of individuals hospitalized for pneumonia in the other regions was obtained by applying an adjustment that took into account health care seeking for acute respiratory illness (ARI) in each region as compared to the base region.

$$I_{NH,Y}=\left( I_{H,Y} \times\frac{1}{{HUS}_{Y}} \right)- I_{H,Y}$$

Equation 4

Where:

$I_{NH,Y}$ = Incidence of non-hospitalized SARI in region Y

${HUS}_{Y}$ = Proportion of all SARI cases that are hospitalized in region Y

$${HUS}_{Y}={HUS}_{B}\times\frac{{DHS}_{Y}}{{DHS}_{B}}$$

Equation 5

Where:

${HUS}_{B}$ = Proportion of all SARI cases that are hospitalized in the base region

${HUS}_{Y}$ = Proportion of all SARI cases that are hospitalized in region Y

1. The percentage of SARI due to influenza in each of the regional influenza surveillance sites was applied to the regional adjusted rate of hospitalized SARI, to obtain the region-specific rates of hospitalized influenza-associated SARI. As the annual number of specimens was limited, the percent positivity of SARI due to influenza was only calculated in two broad age groups: children <5 years and persons aged ≥5 years. The percent positivity for children aged <5 years was applied to all age groups aged <5 years. Similarly, the percent positivity for influenza for persons aged ≥5 years was applied to all age groups five years and older.

$${IF}_{H,Y}=I_{H,Y} \times F_{Y}$$

Equation 6

Where:

${IF}_{H,Y}$ = Incidence of hospitalized influenza-associated SARI in region Y

$F_{Y}$ = Proportion of pneumonia due to influenza

As surveillance data was not available in the Eastern and North Eastern regions over the study period, surveillance data from the sites in the neighboring regions were used to estimate the average proportion of hospitalized influenza-associated SARI that was assigned to these two regions. For the Eastern region, an average proportion of influenza positivity from the Coast, Rift Valley, Central and Nairobi regions was used, while for North Eastern region an average proportion of influenza positivity from Eastern and Coast regions was used.

1. The region specific rates of non-hospitalized influenza-associated SARI were calculated by multiplying the incidence of non-hospitalized SARI by the percent positivity for influenza at each surveillance site.

$${IF}_{NH,Y}=I_{NH,Y} \times{IF}_{,Y}$$

Equation 7

Where:

${IF}_{NH,Y}$ = Incidence of non-hospitalized influenza-associated SARI in region Y

$F_{Y}$ = Proportion of pneumonia due to influenza

1. To obtain the number of hospitalized and non-hospitalized cases of SARI and influenza-associated SARI, the rates in each region were multiplied with the regional population data.

$${NI}_{H,Y}=I_{H,Y} \times P{op}_{Y}$$

Equation 8

$${NI}_{NH,Y}=I_{NH,Y} \times P{op}_{Y}$$

Equation 9

$${NF}_{H,Y}={IF}_{H,Y} \times P{op}_{Y}$$

Equation 10

$${NF}_{NH,Y}={IF}_{NH,Y} \times P{op}_{Y}$$

Equation 11

Where:

${NI}_{H,Y}$ = Number of hospitalized SARI cases in region Y

${NI}_{NH,Y}$ = Number of non-hospitalized SARI cases in region Y

${NF}_{H,Y}$ = Number of hospitalized influenza-associated SARI cases in region Y

${NF}_{NH,Y}$ = Number of non-hospitalized influenza-associated SARI cases in region Y

$P{op}_{Y}$ = Population in region Y

## Differences in methodology between original and current study

There were a few differences between the original method described by Fuller et al. (1) and the current method. In the current study the catchment population of Karemo division was used to determine the base rates of SARI among those enrolled within the HDSS. In the method proposed by Fuller, base rates of SARI were calculated using a catchment population within 5 kilometers (km) of SCRH. However, an exploratory analysis of SARI rates calculated using patients and residents within a 5 km radius of SCRH versus the whole of Karemo division produced similar results. Considering the finer age groups investigated and the potential for recording very few SARI cases for some age groups, the larger population of Karemo was chosen over a subset residing within a 5 km radius of SCRH.

Given the previous lack of data on HIV prevalence in children, Fuller and colleagues had proposed an algorithm for calculation of HIV prevalence in children. This algorithm took into account the prevalence of HIV positive mothers in each region, enrollment in prevention to mother to child transmission programs per region, and the HIV mother to child transmission rates. At the time of the current study, the Kenya HIV Estimates 2014 provided the number of children 0 to 14 years of age living with HIV in each region. By dividing this number with the population of each region, a rough estimate of the prevalence of HIV in children per region was obtained. The national estimate obtained from this approach (1.0%) was similar to published results from the Kenya AIDS Indicator Survey 2012 (0.9%) (3).

Lastly, when calculating the rates of non-hospitalized influenza-associated SARI, health care utilization survey data on the proportion of individuals with pneumonia who were admitted in the past 12 months was used, rather than the proportion of individuals with pneumonia who visited a hospital in the past 12 months as was used in the original study. It was felt that in calculating the rate of non-hospitalized influenza-associated SARI cases, using the proportion who were admitted rather than the proportion who visited a hospital would more accurately reflect the ratio of hospitalized to non–hospitalized cases. It was observed by the current study research team that a visit to the hospital though indicative of access to a health facility as explained in the original Fuller method, would not necessarily equate to hospitalization. Within the Kenyan setting, hospitalization is not only dependent on the severity of illness but also on whether in-patient facilities are available within the health facility, as well as the ability of the patient to afford admission. For these reasons it was argued that the proportion of individuals with pneumonia who were admitted in the past 12 months would more accurately reflect the difference between hospitalized and non-hospitalized influenza cases as compared to the proportion of individuals who visited a hospital. This change in input parameter would change the ratio of hospitalized to non-hospitalized patients. As a result, the rates of non-hospitalized influenza-associated SARI were similar in both study periods. S6 Table provides a summary of the differences in methodology between the original study conducted by Fuller *et al* and the current study and S7 Table presents the risk factors’ relative risk values and the sources of data for both the original and current study.

# References

1. Fuller JA, Summers A, Katz MA, Lindblade KA, Njuguna H, Arvelo W, et al. Estimation of the national disease burden of influenza-associated severe acute respiratory illness in Kenya and Guatemala: a novel methodology. PLoS One. 2013;8(2):e56882.

2. Burton DC, Flannery B, Onyango B, Larson C, Alaii J, Zhang X, et al. Healthcare-seeking behaviour for common infectious disease-related illnesses in rural Kenya: a community-based house-to-house survey. Journal of health, population, and nutrition. 2011;29(1):61-70.

3. Ng'eno B, Mwangi A, Ng'ang'a L, Kim AA, Waruru A, Mukui I, et al. Burden of HIV infection among children aged 18 months to 14 years in Kenya: results from a nationally representative population-based cross-sectional survey. Journal of acquired immune deficiency syndromes (1999). 2014;66 Suppl 1:S82-8.

4. Rudan I, Boschi-Pinto C, Biloglav Z, Mulholland K, Campbell H. Epidemiology and etiology of childhood pneumonia. Bulletin of the World Health Organization. 2008;86(5):408-16.

5. Kenya National Bureau of Statistics, National AIDS Control Council, National AIDS and STI Control Programme, Ministry of Public Health and Sanitation, Kenya Medical research Institute, National Coordinating Agency for Populatioin and Development, et al. Kenya Demographic and Health Survey 2008 - 2009. Nairobi: Government of Kenya; 2010.

6. Kenya National Bureau of Statistics, Ministry of Health Kenya, National AIDS Control Council, Kenya Medical Research Institute, National Council for Population and Development, International TDPI. Kenya Demographic and Health Survey 2014. Nairobi: Government of Kenya; 2015.

7. U.S. Agency for International Development. The DHS Program Demographic and Health Surveys - Data 2016 [Available from: http://www.dhsprogram.com/Data/.

8. Central Bureau of Statistics, MInistry of Finance and Planning. Kenya Multiple Indicator Cluster Survey 2000 Preliminary report. Nairobi: Government of Kenya, UNICEF; 2000.

9. Kenya National Bureau of Statistics. The 2009 Kenya Population and Housing Census. Volume 1C - Population Distribution by Age, Sex and Administrative Units. 2010.

10. Central Bureau of Statistics, Ministry of Health Kenya, Kenya Medical Research Institute, National Council for Population and Development, ORC Macro, Centers for Disease Control and Prevention Nairobi Kenya. Kenya Demographic and Health Survey 2003. Nairobi: Ministry of Health; 2004.

11. Roca A, Sigauque B, Quinto L, Morais L, Berenguera A, Corachan M, et al. Estimating the vaccine-preventable burden of hospitalized pneumonia among young Mozambican children. Vaccine. 2010;28(30):4851-7.

12. National AIDS Control Council, National AIDS and STI Control Programme. Kenya HIV Estimates 2014. Ministry of Health; 2014 2014.

13. Iwuji CC, Mayanja BN, Weiss HA, Atuhumuza E, Hughes P, Maher D, et al. Morbidity in HIV-1-infected individuals before and after the introduction of antiretroviral therapy: a longitudinal study of a population-based cohort in Uganda. HIV medicine. 2011;12(9):553-61.

14. National AIDS and STI Control Programme. Kenya AIDS Indicator Survey 2012. Nairobi: Ministry of Health; 2014.
